# Cancer-associated SF3B1-K700E mutation controls immune responses by regulating T_reg_ function via aberrant *Anapc13* splicing

**DOI:** 10.1126/sciadv.ado4274

**Published:** 2024-09-20

**Authors:** Yun Shi, Wencan Zhang, Qiong Jia, Xiancai Zhong, Prajish Iyer, Hongmin Wu, Yate-Ching Yuan, Yuqi Zhao, Lianjun Zhang, Lili Wang, Zhenyu Jia, Ya-Huei Kuo, Zuoming Sun

**Affiliations:** ^1^ Department of Immunology & Theranostics, Arthur Riggs Diabetes & Metabolism Research Institute, Beckman Research Institute of the City of Hope, Duarte, CA 91010, USA.; ^2^ Department of Botany & Plant Sciences, University of California, Riverside, CA 92527, USA.; ^3^ Department of System Biology, Beckman Research Institute of the City of Hope, Duarte, CA 91010, USA.; ^4^ Translational Bioinformatics, Department of Computational Quantitative Medicine, Beckman Research Institute of the City of Hope, Duarte, CA 91010, USA.; ^5^ Integrated Genomics Core, Beckman Research Institute of the City of Hope, Duarte, CA 91010, USA.; ^6^ Gehr Family Center for Leukemia Research, Department of Hematological Malignancies Translational Science, Hematologic Malignancies and Stem Cell Transplantation Institute, Beckman Research Institute of the City of Hope, Duarte, CA 91010, USA.

## Abstract

Recurrent somatic mutations in spliceosome factor 3b subunit 1 (SF3B1) are identified in hematopoietic malignancies, with SF3B1-K700E being the most common one. Here, we show that regulatory T cell (T_reg_)–specific expression of SF3B1-K700E (*Sf3b1^K700Efl/+^/Foxp3^YFP-Cre^*) results in spontaneous autoimmune phenotypes. CD4^+^ T cells from *Sf3b1^K700Efl/+^/Foxp3^YFP-Cre^* mice display defective T_reg_ differentiation and inhibitory function, which is demonstrated by failed prevention of adoptive transfer colitis by *Sf3b1^K700Efl/+^/Foxp3^YFP-Cre^* T_regs_. Mechanically, SF3B1-K700E induces an aberrant splicing event that results in reduced expression of a cell proliferation regulator *Anapc13* due to the insertion of a 231–base pair DNA fragment to the 5′ untranslated region. Forced expression of the *Anapc13* gene restores the differentiation and ability of *Sf3b1^K700Efl/+^/Foxp3^YFP-Cre^* T_regs_ to prevent adoptive transfer colitis. In addition, acute myeloid leukemia grows faster in aged, but not young, *Sf3b1^K700Efl/+^/Foxp3^YFP-Cre^* mice compared to *Foxp3^YFP-Cre^* mice. Our results highlight the impact of cancer-associated *SF3B1* mutation on immune responses, which affect cancer development.

## Teaser

SF3B1-K700E mutation controls immune responses via alternative *Anapc13* splicing to regulate T_reg_ function.

## INTRODUCTION

Spliceosome factor 3b subunit 1 (SF3B1), a critical component of the core spliceosome U2 small nuclear ribonucleoprotein complex, regulates pre-mRNA splicing (*1*–*3*). Large-scale cancer genome sequencing projects have identified recurrent somatic mutations in SF3B1 in several types of hematological malignancies including chronic lymphocytic leukemia (CLL), acute myeloid leukemia (AML), and myelodysplastic syndromes (MDS) (*4*–*6*). About half of MDS tumor samples have mutations in spliceosome genes, with *SF3B1* being the most commonly mutated one (*7*, *8*). Several lines of evidence support that *SF3B1* mutations often represent founding genetic lesions and thus are major determinants of disease phenotype and have independent prognostic values on survival and risk of progression to AML (*6*, *9*–*12*). The most common *SF3B1* mutation is an A to G transition that results in lysine to glutamic acid substitution at amino acid position 700 (SF3B1-K700E) (*4*, *13*). Previous studies have focused on how mutant *SF3B1* intrinsically promotes the development of cancers via aberrant RNA splicing. For example, it has been demonstrated that the *SF3B1-K700E* mutation alters splicing events, resulting in the dysregulation of multiple cellular pathways including DNA damage and telomere maintenance pathways that drive oncogenesis (*14*–*16*). However, *SF3B1* mutations can be traced back to earlier hematopoietic cells that differentiate into different types of immune cells including regulatory T cells (T_regs_). It largely remains unknown how *SF3B1* mutation affects the function of T_regs_ that plays an important role in controlling the scale of immune responses.

T_regs_ are required to protect against autoimmune responses, maintain homeostasis, and dampen immune responses after clearance of infection (*17*). The important physiological function of T_regs_ for induction and maintenance of peripheral tolerance is illustrated by the uncontrollable autoinflammation in mice and humans that lack functional T_regs_ due to a mutation in the Forehead Box P3 (Foxp3) gene (*18*–*20*). Foxp3 is a lineage-specific transcription factor that regulates the generation, maintenance, and function of T_regs_ (*21*). Natural T_regs_ (nT_regs_) develop in the thymus (or called tT_regs_) mostly with T cell receptors recognizing self-antigens (*22*, *23*), whereas induced T_regs_ (iT_reg_) are differentiated from activated naive CD4^+^ T cells in the presence of transforming growth factor–β (TGF-β) (*21*, *24*). In addition to T_reg_, naive CD4^+^ T cells also differentiate into inflammatory T cells including T helper 1 (T_H_1), T_H_2, and T_H_17 (*25*, *26*), which are inhibited by T_regs_. A fine-tuned balance between inflammatory T cells and T_regs_ is essential for a functional immune system. Skewing to inflammatory T cells leads to autoimmunity, whereas immune tolerance is induced by the dominance of T_regs_. Thus, understanding the mechanisms that regulate the differentiation and function of T_regs_ facilitates the development of novel immunotherapies for controlling immune responses.

Considering the importance of T_regs_ in controlling immune responses, we determined how hematological malignancy-associated Sf3b1 mutation, Sf3b1-K700E in this case, affects the T_regs_ by using mice that express Sf3b1-K700E specifically in T_regs_ (*Sf3b1^K700Efl/+^/Foxp3^YFP-Cre^*). *Sf3b1^K700Efl/+^/Foxp3^YFP-Cre^* mice displayed autoimmune phenotypes including splenomegaly and infiltration of lymphocytes including interferon-γ (IFN-γ)–producing T cells to tissues such as lung and liver. Upon induction, *Sf3b1^K700Efl/+^/Foxp3^YFP-Cre^* mice developed aggravated experimental autoimmune encephalomyelitis (EAE), which was associated with a reduced number of T_regs_. CD4^+^ T cells were defective in T_reg_ differentiation. In addition, *Sf3b1^K700Efl/+^/Foxp3^YFP-Cre^* T_regs_ had greatly impaired inhibitory function in vitro in inhibiting T cell activation and in vivo in preventing colitis induced by adoptive transfer of naive CD4^+^ T cells. We identified an aberrant splicing event at the *Anapc13* gene induced by Sf3b1-K700E expression that accounted for the impaired T_reg_ differentiation and function observed in *Sf3b1^K700Efl/+^/Foxp3^YFP-Cre^* mice. The Sf3b1-K700E–induced aberrant splicing event resulted in the insertion of a DNA fragment to the 5′ untranslated region (5′UTR) of the *Anapc13* gene, which greatly reduced *Anapc13* expression. On the other hand, forced expression of *Anapc13* in *Sf3b1^K700Efl/+^/Foxp3^YFP-Cre^* T_regs_ restored the differentiation and function of T_regs_. In addition, transplanted AML cells grew faster in aged, but not young, *Sf3b1^K700Efl/+^/Foxp3^YFP-Cre^* mice. Our results thus highlight the vital impact of this hematological malignancy-associated mutation on immune tolerance and cancer development.

## RESULTS

### Sf3b1-K700E mutant impairs T_reg_ differentiation

To determine whether and how Sf3b1-K700E mutation affects T_regs_, we used knock-in *Sf3b1^K700Efl/+^* mice in which Cre induces the expression of Sf3b1-K700E from the chromosome containing the knock-in allele, whereas the wild-type (WT) Sf3b1 is expressed from the other chromosome (*14*, *27*), as such mutation is often heterozygous. *Sf3b1^K700Efl/+^* mice were crossed to *Foxp3^YFP-Cre^* mice or *CD4^Cre^* mice to express Sf3b1-K700E only in T_regs_ (*Sf3b1^K700Efl/+^/Foxp3^YFP-Cre^* mice) or T cells (*Sf3b1^K700Efl/+^/CD4*^*C*re^ mice) (fig. S1A for genotyping WT and knock-in allele). Sequencing analysis confirmed K700 (AAA) to E (GAA) mutation (fig. S1B). Two peaks observed at the mutated nucleotide indicate the mixture of A from the WT chromosome and mutated nucleotide G from the chromosome containing the knock-in allele.

**Fig. 1.**
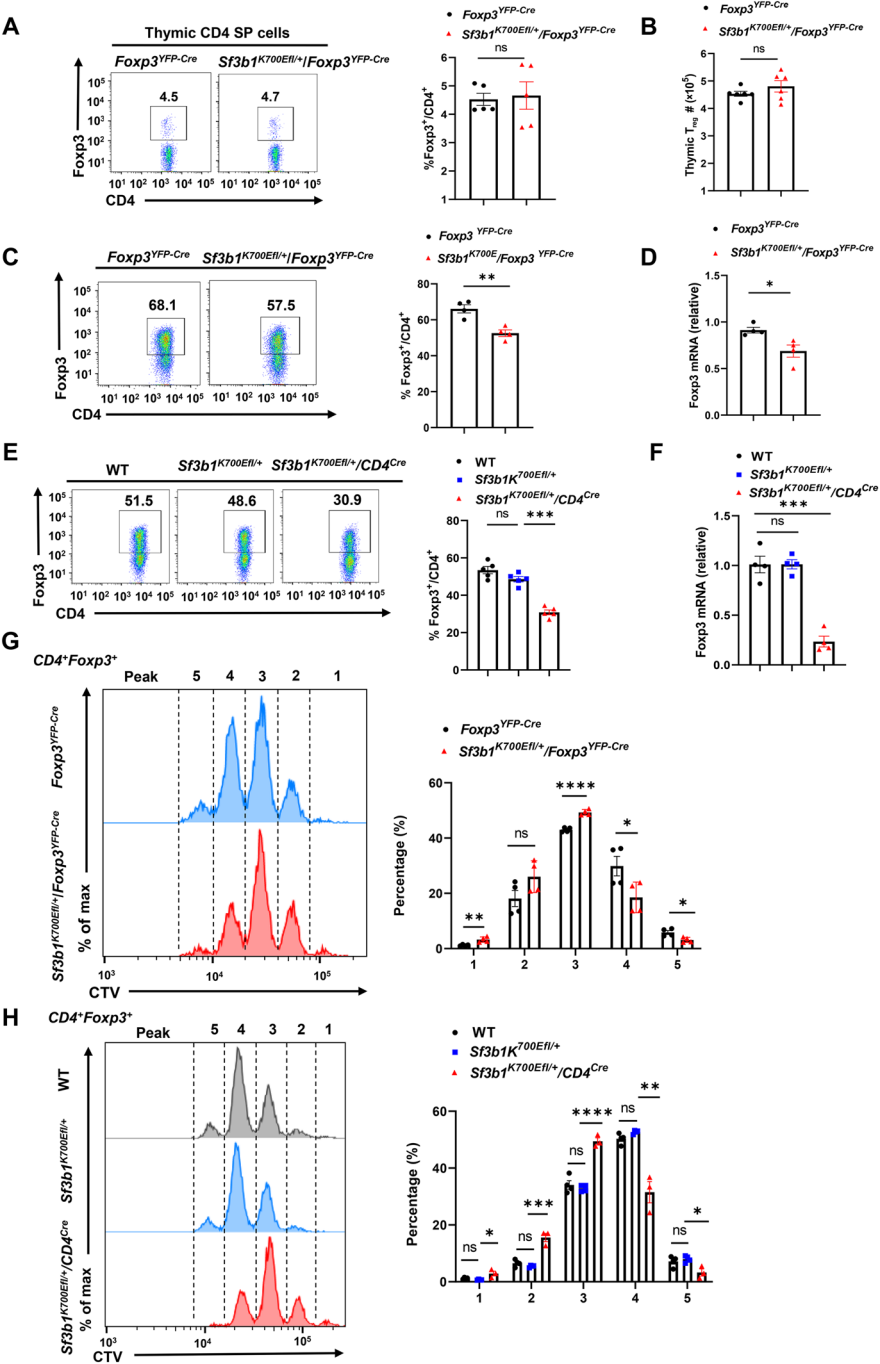
Sf3b1-K700E mutant impairs T_reg_ differentiation. (**A** and **B**) Representative flow cytometric analysis [(A), left], the percentage [(A), right], and number (B) of T_reg_ (Foxp3^+^) cells in thymocytes from indicated mice (*n* ≥ 4 per genotype). (**C** and **D**) Representative flow cytometric analysis [(C), left] and percentage [(C), right] of Foxp3^+^ T_regs_ differentiated from *Foxp3^YFP-Cre^* or *Sf3b1^K700EFl/+^/Foxp3^YFP-Cre^* naive CD4^+^ T cells stimulated in the presence of TGF-β (5 ng/ml) for 48 hours (*n* ≥ 4 per treatment cohort). *Foxp3* mRNA levels in differentiated T_regs_ shown in (C) were detected by quantitative polymerase chain reaction (qPCR) (D). (**E** and **F**) Representative flow cytometric analysis [(E), left] and percentage [(E), right] of Foxp3^+^ T_regs_ differentiated from naive WT, *Sf3b1^K700EFl/+^*, and *Sf3b1^K700EFl/+^/CD4^Cre^* CD4^+^ T cells stimulated in the presence of TGF-β ( 5 ng/ml) for 48 hours (*n* ≥ 4 per treatment cohort). Foxp3 mRNA levels in differentiated T_regs_ shown in (E) were detected by qPCR (F). (**G**) Representative flow cytometric analysis (left) and percentage (right) of the proliferating *Foxp3^YFP-Cre^* or *Sf3b1^K700EFl/+^/Foxp3^YFP-Cre^* CD4^+^ T cells in indicated peak shown on left, labeled with CellTrace Violet (CTV), polarized under T_reg_ conditions for 48 hours (*n* ≥ 4 per genotype). (**H**) Representative flow cytometric analysis (left) and percentage (right) of the proliferative dye–labeled WT, *Sf3b1^K700EFl/+^*, and *Sf3b1^K700EFl/+^/CD4^Cre^* CD4^+^ T cells in indicated peak shown on left, labeled with CTV, polarized under T_reg_ conditions for 48 hours (*n* ≥ 4 per genotype). Boxed area: cell population of interest. Data are from three experiments [(B), (D), and (F); (A), (C), (E), and (G) to (I), right panels, presented as means ± SEM] or are from one representative of three independent experiments [(A), (C), (E), (G), and (H), left panels]. *P < 0.05, **P < 0.01, ***P < 0.001, and ****P < 0.0005; ns, not significant (two-tailed Student’s *t* test).

Since T_regs_ develop in the thymus (tT_regs_), thymic T_reg_ development was first examined. Thymocyte development was overall normal in *Sf3b1^K700Efl/+^/Foxp3^YFP-Cre^* mice, indicated by equivalent thymic cellularity (fig. S1C) and percentage of different developmental stages of thymocyte subsets: CD4^−^CD8^−^ double-negative (early thymocytes), CD4^+^CD8^+^ double-positive, and CD4^+^/CD8^+^ single-positive (mature T cells) cells when compared to *Foxp3^YFP-Cre^* mice (fig. S1D). There was no notable difference in the percentage and the number of thymic T_regs_ between *Foxp3^YFP-Cre^* and *Sf3b1^K700Efl/+^/Foxp3^YFP-Cre^* mice (Fig. 1, A and B). We further monitored Helios and Nrp-1 expression that was reported to be up-regulated on tT_regs_ (*28*, *29*) and did not observe the obvious difference in their expression on T_regs_ from *Foxp3^YFP-Cre^* and *Sf3b1^K700Efl/+^/Foxp3^YFP-Cre^* spleens (fig. S1E). Thus, our results do not support that Sf3b1-K700E mutation obviously affects T_reg_ development in the thymus.

iT_regs_ are differentiated from peripheral naive CD4^+^ T cells in the presence of TGF-β. We next examined how Sf3b1-K700E mutation affects iT_reg_ differentiation. We confirmed that purified naive CD4^+^ T cells from spleens of *Foxp3^YFP-Cre^* and *Sf3b1^K700Efl/+^/Foxp3^YFP-Cre^* mice were Foxp3^−^ (fig. S1F), whereas these naive CD4^+^ T cells differentiated into Foxp3^+^ T_regs_ when activated in the presence of TGF-β (Fig. 1C). However, the ability of CD4^+^ T cells from *Sf3b1^K700Efl/+^/Foxp3^YFP-Cre^* mice to generate iT_regs_ was impaired compared to the CD4^+^ T cells from *Foxp3^YFP-Cre^* mice. Consistently, *Foxp3* mRNA was decreased in *Sf3b1^K700Efl/+^/Foxp3^YFP-Cre^* CD4^+^ T cells compared to that of *Foxp3^YFP-Cre^* CD4^+^ T cells after T_reg_ differentiation (Fig. 1D). The observed impaired T_reg_ differentiation was not due to changes in cell survival, which were comparable between *Foxp3^YFP-Cre^* and *Sf3b1^K700Efl/+^/Foxp3^YFP-Cre^* cells (fig. S1G). The impaired iT_reg_ differentiation together with reduced *Foxp3* mRNA was also confirmed using naive CD4^+^ T cells (fig. S1H) from *Sf3b1^K700Efl/+^/CD4*^*C*re^ mice that displayed more marked defects than that observed with *Sf3b1^K700Efl/+^/Foxp3^YFP-Cre^* CD4^+^ T cells (Fig. 1, E and F). This is likely due to that the expression of the Sf3b1-K700E mutant in *Sf3b1^K700Efl/+^/CD4*^*C*re^ CD4^+^ T cells was before the induction of T_reg_ differentiation, whereas *Sf3b1^K700Efl/+^/Foxp3^YFP-Cre^* CD4^+^ T cells started to express the Sf3b1-K700E mutant after T_reg_ differentiation when Foxp3 was induced. We next monitored cell proliferation during T_reg_ differentiation and found that the proliferation of *Sf3b1^K700Efl/+^/Foxp3^YFP-Cre^* T_regs_ was slower than that of *Foxp3^YFP-Cre^* T_regs_ (Fig. 1G). Consistently, reduced proliferation was also observed in *Sf3b1^K700Efl/+^/CD4*^*C*re^ T_regs_ (Fig. 1H). However, the defective proliferation was not observed in stimulated purified T_regs_ (fig. S1I). Therefore, the Sf3b1-K700E mutant impairs the differentiation of iT_regs_ by reducing proliferation.

### Sf3b1-K700E mutant impairs the generation of iT_reg_ in vivo

**Fig. 2.**
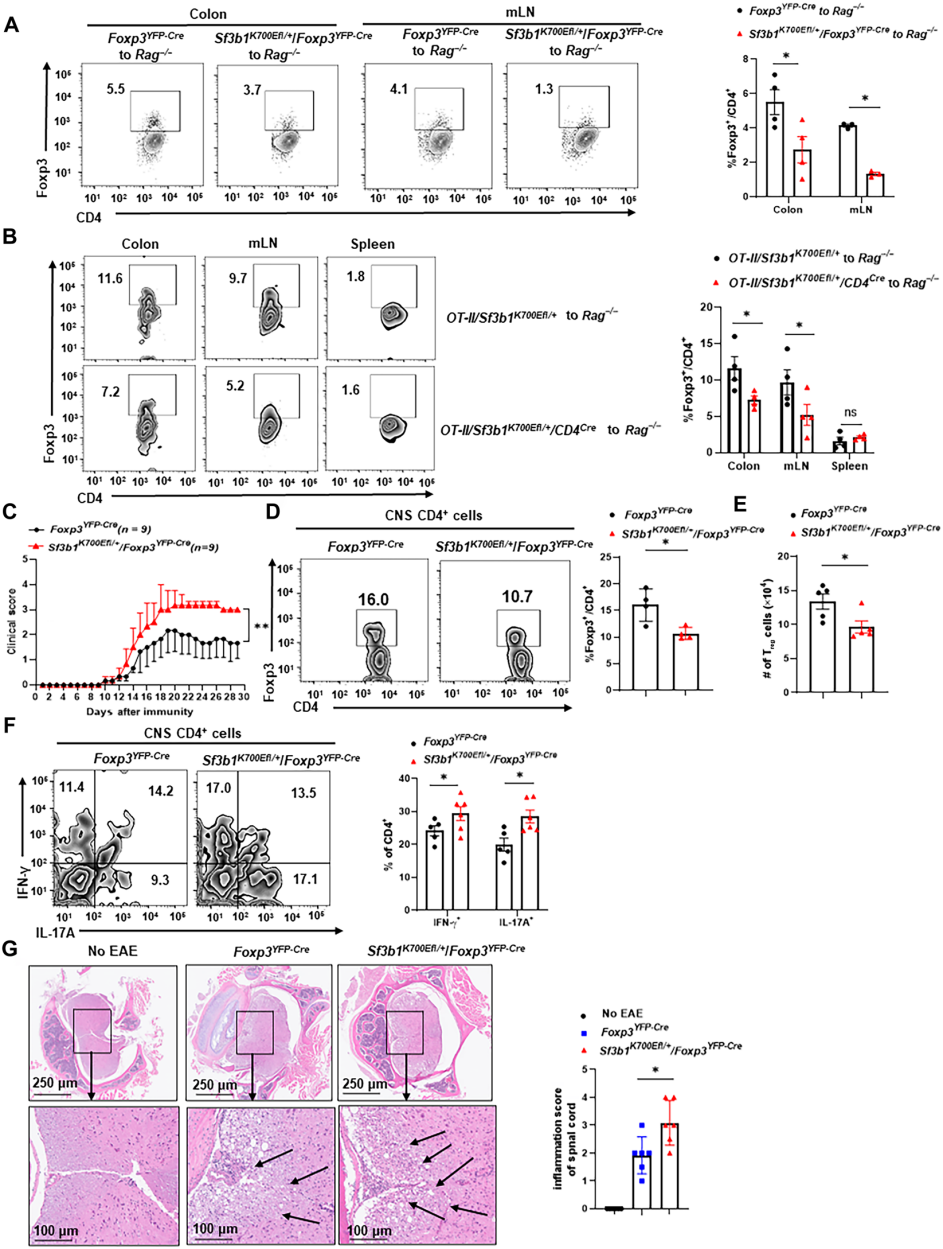
Sf3b1-K700E mutant impairs the generation of iT_reg_ in vivo. (**A**) Representative flow cytometric analysis (left) and percentage (right) of Foxp3^+^CD4^+^ T_regs_ in the colon and mesenteric lymph nodes (mLNs) 3 weeks after adoptive transfer of indicated 0.4 × 10^6^ naive CD4^+^ T cells to *Rag1*^−/−^ mice (*n* ≥ 4 per genotype). (**B**) Representative flow cytometric analysis (left) and percentage (right) of Foxp3^+^CD4^+^ T_regs_ in colon, mLN, and spleen of *Rag1*^−/−^ mice transferred with 3 × 10^6^ naive *OT-II/ Sf3b1^K700Efl/+^* or *OT-II/ Sf3b1^K700Efl/+^/CD4^Cre^* naive CD4^+^ T cells and subsequently fed with ovalbumin (OVA)-containing drinking water (20 mg/ml) for 5 days (*n* ≥ 4 per genotype). (**C**) Mean clinical EAE scores of indicated mice at different days after EAE induction with MOG_35–55_ (*n* = 9 per genotype). (**D** and **E**) Representative flow cytometric analysis [(D), left], the percentage [(D), right], and number (E) of Foxp3^+^CD4^+^ T_regs_ recovered from the central nervous system (CNS) of EAE-induced mice shown in (C) (*n* ≥ 5 per genotype). (**F**) Representative flow cytometric analysis (left) and the percentage (right) of IFN-γ^+^ and interleukin-17A–positive (IL-17A^+^) cells among CD4^+^ T cells recovered from the CNS of EAE-induced mice shown in (C) (*n* ≥ 7 per genotype). (**G**) Section of hematoxylin and eosin (H&E)–stained spinal cord from post-EAE induction in indicated mice shown in (C) (left). The right panel is the inflammation score based on observed lymphocyte infiltration shown on the left panels (*n* ≥ 4 per genotype). Boxed area: cell population of interest. Data are from three experiments [(D), presented as means ± SEM; (B), (C), (E), and (G), right panels, presented as means ± SEM] or are from one representative of three independent experiments [(A); (B), (C), (E), and (G), left panels]. *P < 0.05 (two-tailed Student’s *t* test).

To determine whether the Sf3b1-K700E mutant affects the T_reg_ differentiation in vivo, sorted naive *Foxp3^YFP-Cre^* or *Sf3b1^K700Efl/+^/Foxp3^YFP-Cre^* CD4^+^ T cells that lack Foxp3^+^ T_regs_ (fig. S1F) were adoptively transferred to *Rag1*^−/−^ mice (*30*, *31*). T_regs_ were detected in spleens and mesenteric lymph nodes (mLNs) 3 weeks after the adoptive transfer of naive *Foxp3^YFP-Cre^* CD4^+^ T cells (Fig. 2A). However, naive *Sf3b1^K700Efl/+^/Foxp3^YFP-Cre^* CD4^+^ T cells generated much fewer T_regs_ in vivo. Next, an oral tolerance model was used to determine the effects of Sf3b1-K700E on the generation of T_regs_ in vivo (*24*). In this model, sorted naive CD4^+^ T cells from *OT-II/Sf3b1^K700Efl/+^* or *OT-II*/ *Sf3b1^K700Efl/+^/CD4^Cre^* mice were adoptively transferred into *Rag1*^−/−^ mice, and a notable amount of T_regs_ was induced mostly in gut-associated lymphoid tissues such as the colon and mLNs, but not the spleen, by orally administrated ovalbumin (OVA) peptide in drinking water (Fig. 2B). Consistently, much less T_regs_ were induced from *OT-II*/*Sf3b1^K700Efl/+^/CD4^Cre^* CD4^+^ T cells than that from *OT-II/Sf3b1^K700Efl/+^* CD4^+^ T cells. Collectively, these results demonstrate that the Sf3b1-K700E mutant impairs T_reg_ differentiation in vivo.

To determine whether the Sf3b1-K700E mutant–impaired generation of T_regs_ affects immune responses in vivo, we compared the development of EAE between *Foxp3^YFP-Cre^* and *Sf3b1^K700Efl/+^/Foxp3^YFP-Cre^* mice. Compared to *Foxp3^YFP-Cre^* mice, *Sf3b1^K700Efl/+^/Foxp3^YFP-Cre^* mice developed much severe EAE together with more weight loss (Fig. 2C and fig. S2A) and had less number and percentage of T_regs_ (Fig. 2, D and E), whereas more inflammatory CD4^+^IFN-γ^+^ and CD4^+^IL-17A^+^ (interleukin-17A–positive) cells in the central nervous system (CNS) (Fig. 2F and fig. S2B for gating strategy). Consistently, the histochemical examination also observed that *Sf3b1^K700Efl/+^/Foxp3^YFP-Cre^* mice had more infiltrated lymphocytes to CNS and tissue damages with a much higher inflammation score (Fig. 2G). Our results thus demonstrate that the Sf3b1-K700E mutant impairs T_reg_ differentiation in vivo, which leads to aggravated immune responses for induction of EAE.

### T_regs_ from *Sf3b1^K700Efl/+^/Foxp3^YFP-Cre^* mice have impaired inhibitory function

**Fig. 3.**
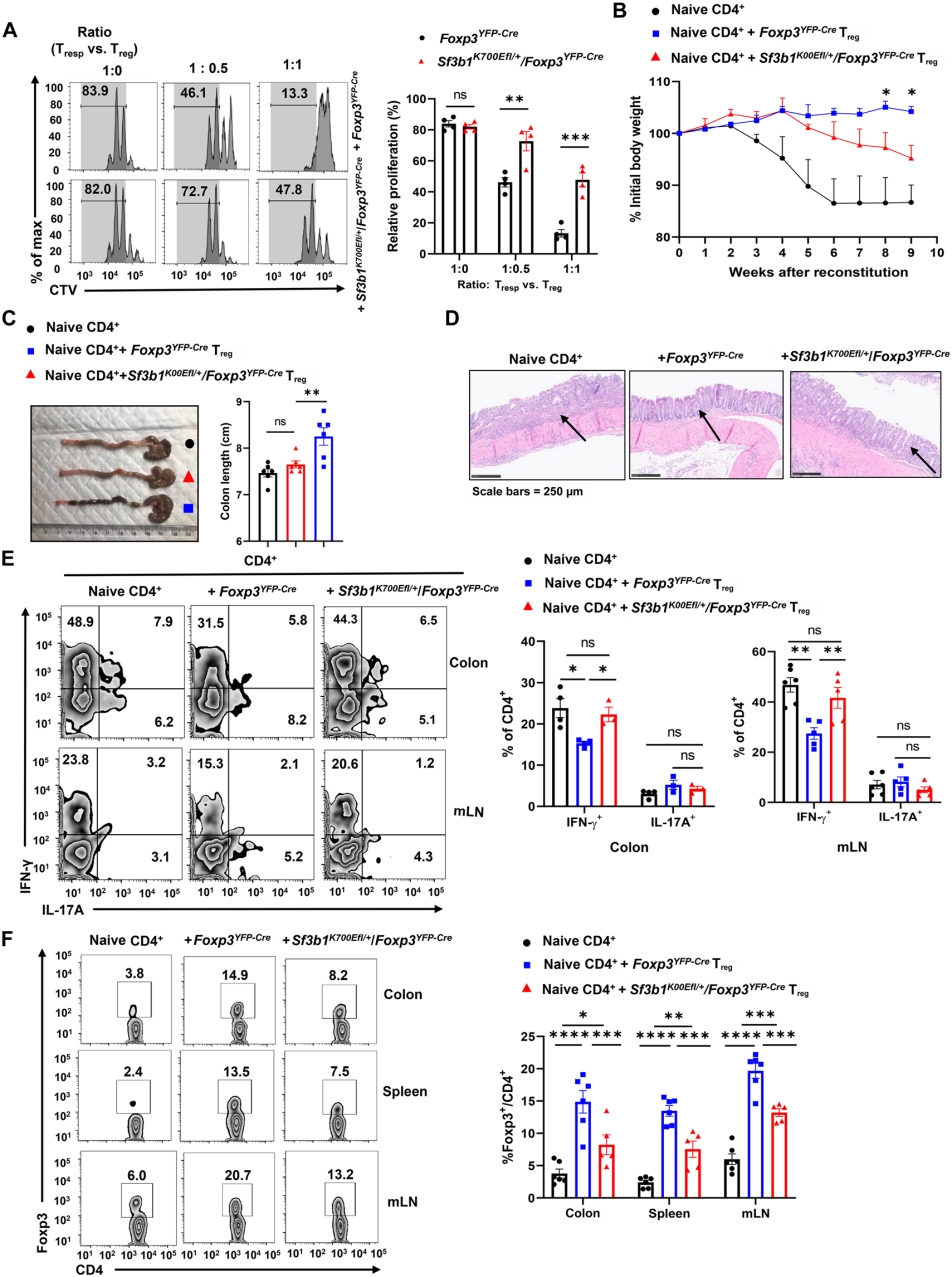
T_regs_ from *Sf3b1^K700Efl/+/^Foxp3^YFP-Cre^* mice have impaired inhibitory function. (**A**) Representative flow cytometric analysis (left) and percentage (right) of the proliferated responder CD4^+^ T cells (T_resp_) in the gated area shown on left, labeled with CTV dye and cocultured with splenic YFP^+^CD4^+^ T_regs_ isolated from indicated 6- to 8-week-old mice (*n* ≥ 4 per genotype). (**B**) Body weight of *Rag1*^−/−^ recipients over time after adoptive transfer of naive WT CD45RB^hi^CD25^−^CD4^+^ T cells alone or in combination with purified splenic T_regs_ from indicated 6- to 8-week-old mice. (**C**) Representative image of colons (left) and colon length (right) (*n* ≥ 5 per genotype) from colitis-induced mice shown in (B). (**D**) H&E-stained colon section from colitis-induced recipients shown in (B) 9 weeks after adoptive transfer. (**E**) Representative flow cytometric analysis (left) and percentage (right) of CD4^+^IL-17A^+^ and CD4^+^IFN-γ^+^ cells recovered from colons or mLN of colitis-induced recipients shown in (B) (*n* ≥ 4 per group). (**F**) Representative flow cytometric analysis (left) and percentage (right) of Foxp3^+^CD4^+^ T_regs_ recovered from colon, spleen, and mLN of colitis-induced recipients shown in (B) (*n* ≥ 4 per group). Boxed area: cell population of interest. Data are from three experiments [(A) to (C), (E), and (F), right panels, presented as means ± SEM] or are from one representative of three independent experiments [(B); (A), (C), (D), and (F), left panels]. *P < 0.05, **P < 0.01, ***P < 0.001, and ****P < 0.0005 (two-tailed Student’s *t* test).

In addition to T_reg_ differentiation, the ability of T_reg_ to suppress T cell activation also controls the scale of immune responses. We first examined the expression of several surface markers, CD25, CD73, CD39, and CTLA-4 (fig. S3A) that are the indicators for the suppressive function of T_regs_ (*32*–*34*). *Sf3b1^K700Efl/+^/Foxp3^YFP-Cre^* T_reg_ had lower levels of CD25, CD73, and Foxp3, already indicating impaired suppressive function. We next assessed the ability of T_regs_ to suppress CD4^+^ T cell proliferation in vitro. In vitro differentiated iT_regs_ from *Sf3b1^K700Efl/+^/Foxp3^YFP-Cre^* mice showed greatly impaired suppressive function in inhibiting CD4^+^ T cell proliferation compared to the T_regs_ from *Foxp3^YFP-Cre^* mice (Fig. 3A and fig. S3B). We also sorted YFP^+^Nrp-1^−^ iT_reg_ directly from mice to determine their ability to suppress CD4^+^ T cell proliferation (fig. S3C). Consistently, iT_reg_ from *Sf3b1^K700Efl/+^/Foxp3^YFP-Cre^* mice had reduced inhibitory activity compared to iT_reg_ from *Foxp3^YFP-Cre^* mice. Next, the in vivo suppressive function of T_regs_ was examined in the prevention of adoptive transfer colitis. In the absence of T_regs_, adoptive transfer of naive CD4^+^ T cells (CD45RB^hi^CD25^−^CD4^+^) into *Rag1*^−/−^ mice induced severe colitis, as indicated by weight loss (Fig. 3B), shortened colon (Fig. 3C), damaged tissues (Fig. 3D), and greatly increased pro-inflammatory IFN-γ^+^CD4^+^ T, but not IL-17A^+^CD4^+^, cells in the colon and mLN (Fig. 3E), whereas cotransfer of *Foxp3^YFP-Cre^* T_regs_, but not *Sf3b1^K700Efl/+^/Foxp3^YFP-Cre^* T_regs_, prevented these severe colitis phenotypes in *Rag1*^−/−^ mice (Fig. 3, B to E). Furthermore, a lower percentage of T_regs_ were detected in the recipients adoptively transferred with *Sf3b1^K700Efl/+^/Foxp3^YFP-Cre^* T_reg_ compared to the recipients with *Foxp3^YFP-Cre^* T_regs_ in spleen and gut-associated tissues including colon and mLN (Fig. 3F), thus contributing to the observed much severe colitis. Similarly, adoptive transfer of T_regs_ from *Sf3b1^K700Efl/+^/CD4^Cre^* mice also failed to prevent colitis to the levels by T_regs_ from control *Sf3b1^K700Efl/+^* mice, indicated by more weight loss (fig. S3D), a shorter colon (fig. S3E), and a higher number of pro-inflammatory IFN-γ^+^CD4^+^ T, but not IL-17A^+^CD4^+^, cells recovered from the colon (fig. S3F), whereas there were fewer T_regs_ in the spleen, colon, and mLN in *Rag1*^−/−^ recipients with *Sf3b1^K700Efl/+^/CD4^Cre^* T_regs_ (fig. S3G). Their results suggest that the Sf3b1-K700E mutant impairs the suppressive function of T_regs_ in addition to T_reg_ differentiation.

### *Sf3b1^K700Efl/+^/Foxp3^YFP-Cre^* mice develop autoinflammation

**Fig. 4.**
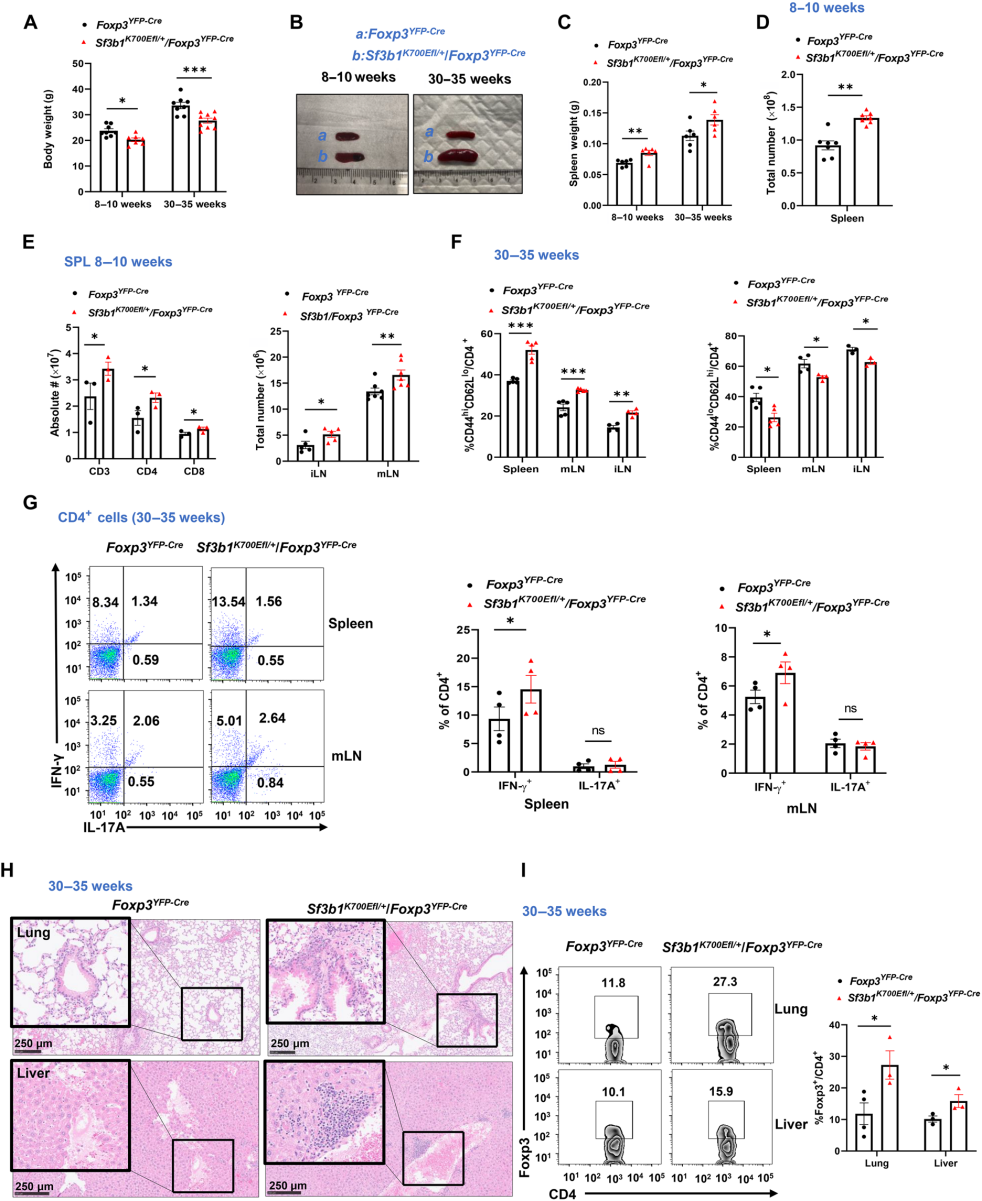
*Sf3b1^K700Efl/+/^Foxp3^YFP-Cre^* mice develop autoinflammation. (**A**) Body weight of indicated 8- to 10-week-old or 30- to 35-week-old mice (*n* ≥ 5 per genotype per group). (**B**) Image of spleens from indicated 8- to 10-week-old (left) or 30- to 35-week-old mice (right). (**C**) Weight of the spleens from indicated 8- to 10-week-old or 30- to 35-week-old mice (*n* ≥ 4 per genotype per group). (**D** and **E**) Total number of cells (D) and CD3^+^, CD4^+^, and CD8^+^ T cells [(E), left] of spleens or inguinal lymph nodes (iLN) or mLNs [(E), right] from indicated mice (*n* ≥ 4 per genotype per group). (**F**) Percentage of CD44^hi^CD62^lo^ memory-like (left) and CD44^lo^CD62^hi^ (right) naive cells among splenic CD4^+^ T cells from indicated aged mice (*n* ≥ 4 per genotype per group). (**G**) Representative flow cytometric analysis (left) and percentage (right) of IL-17A^+^ and IFN-γ^+^ cells among CD4^+^ T cells from spleens or mLN of indicated aged mice (*n* ≥ 4 per genotype). (**H**) Section of H&E-stained lung and liver from indicated 30- to 35-week-old mice. (**I**) Representative flow cytometric analysis (left) and percentage (right) of T_regs_ (Foxp3^+^) among CD4^+^ T cells recovered from lung and liver of 30- to 35-week-old mice (*n* ≥ 4 per genotype). Boxed area: cell population of interest. Data are from three experiments [(A) and (C) to (F); (G) and (I), right panels, presented as means ± SEM] or are from one representative of three independent experiments [(B) and (H); (G) and (I), left panels].*P < 0.05, **P < 0.01, and ***P < 0.001 (two-tailed Student’s *t* test).

We noticed that aged *Sf3b1^K700Efl/+^/Foxp3^YFP-Cre^* mice were smaller indicated by the lighter weight (Fig. 4A) and suffered from dermatitis associated with hair loss (fig. S4A) compared to their *Foxp3^YFP-Cre^* counterparts. Histochemical analysis of the skin found notable lymphocyte infiltration in the skin in *Sf3b1^K700Efl/+^/Foxp3^YFP-Cre^* mice (fig. S4B). *Sf3b1^K700Efl/+^/Foxp3^YFP-Cre^* mice had enlarged spleen compared to *Foxp3^YFP-Cre^* mice (Fig. 4B, left), and the difference in spleen size was increased with aging (Fig. 4B, right). The increased spleen size was also reflected by increased spleen weight (Fig. 4C) and cellularity (Fig. 4D) including CD3^+^ T cells due to an increase in both CD4^+^ and CD8^+^ T cells (Fig. 4E, left). In addition, lymph nodes in *Sf3b1^K700Efl/+^/Foxp3^YFP-Cre^* mice were also larger, indicated by increased cellularity (Fig. 4E, right). These phenotypes indicate that *Sf3b1^K700Efl/+^/Foxp3^YFP-Cre^* mice develop spontaneous autoinflammation. Consistently, analysis of CD4^+^ T cells indicated that there were more CD44hiCD62Llo memory-like, whereas there were reciprocally less CD44loCD62Lhi naive cells both in 8- to 10-week-old (fig. S4C) and 30- to 35-week-older *Sf3b1^K700Efl/+^/Foxp3^YFP-Cre^* mice compared to *Foxp3^YFP-Cre^* mice (Fig. 4F). Furthermore, more IFN-γ^+^, but not IL-17A^+^, inflammatory CD4^+^ T cells were detected in spleens and lymph nodes of older *Sf3b1^K700Efl/+^/Foxp3^YFP-Cre^* mice than that of *Foxp3^YFP-Cre^* mice (Fig. 4G). Increased IFN-γ^+^ CD4^+^ T cells were also observed in younger *Sf3b1^K700Efl/+^/Foxp3^YFP-Cre^* mice (fig. S4D). Histological analysis of lung and liver found notable lymphocyte infiltration together with tissue damage in *Sf3b1^K700Efl/+^/Foxp3^YFP-Cre^* mice (Fig. 4H), similar to that observed in the skin (fig. S4B). Increased percentage and/or number of T_regs_ were observed in the spleen, lymph node, lung, and liver of *Sf3b1^K700Efl/+^/Foxp3^YFP-Cre^* mice (Fig. 4I and fig. S4, E and F). However, these *Sf3b1^K700Efl/+^/Foxp3^YFP-Cre^* T_regs_ had substantially reduced mean fluorescence intensity for *Foxp3* compared to *Foxp3^YFP-Cre^* T_regs_ (fig. S4G). The increased T_regs_ in *Sf3b1^K700Efl/+^/Foxp3^YFP-Cre^* mice are likely a compensatory mechanism trying to inhibit the observed inflammation. This is consistent with what was observed in other mice with defective T_regs_ (*35*, *36*). These results suggest that T_reg_-specific expression of the Sf3b1-K700E mutant breaks the immune tolerance and leads to the development of autoinflammation.

### Sf3b1-K700E mutant induced aberrant splicing events during T_reg_ differentiation

**Fig. 5.**
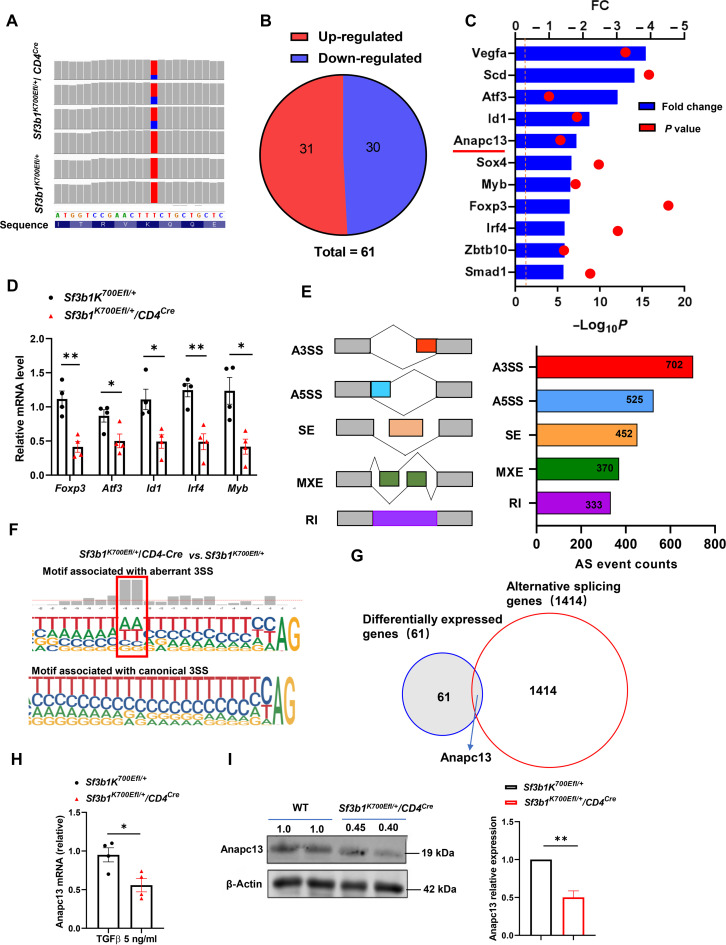
Sf3b1-K700E mutant induced aberrant splicing events during T_reg_ differentiation. (**A**) Sequence fragment density around Sf3b1-K700E from RNA-seq performed with iT_reg_ differentiated from naive *Sf3b1^K700Efl/+^/CD4^Cre^* and *Sf3b1^K700Efl/+^* CD4^+^ T cells in the presence of TGF-β (5 ng/ml) for 48 hours. Red denotes WT (adenine) and blue denotes mutant (guanine) nucleotide reads. Mutant allele frequency ranged from 22.9 to 38.7%. (**B**) Venn diagram of a number of differentially expressed genes between *Sf3b1^K700Efl/+^* and *Sf3b1^K700Efl/+^/CD4^Cre^* iT_reg_ (*n* = 3 per genotype). Up-regulated genes (red) and down-regulated genes (blue) in *Sf3b1^K700Efl/+^/CD4^Cre^* iT_reg_ with a cutoff at *P* < 0.05 and fold change |FC| ≥ 1.5 are shown. (**C**) A list of down-regulated genes in *Sf3b1^K700Efl/+^/CD4^Cre^* iT_reg_ cells. (**D**) qPCR analysis of *Foxp3*, *Atf3*, *Id1*, *Myb*, and *Irf4* mRNA in indicated iT_reg_ cells (*n* ≥ 4 per genotype per group). (**E**) The number and indicated types of alternative splicing events in *Sf3b1^K700Efl/+^/CD4^Cre^* iT_reg_ cells, identified by the alignment of RNA-seq sequence with genomic DNA sequence (three mice per group; false discovery rate < 0.1). (**F**) Motif frequency plots for canonical and aberrant 3′ splice site (3SS). The motifs showing are 35 nucleotides (nt) upstream of the 30 AG and 2 nt downstream. (**G**) Venn diagram of gene overlapping between 61 differentially expressed genes and 1414 alternative splicing genes between *Sf3b1^K700Efl/+^* and *Sf3b1^K700Efl/+^/CD4^Cre^* iT_reg_. (**H**) qPCR analysis of *Anapc13* mRNA levels in indicated CD4^+^ T cells polarized under T_reg_ conditions for 48 hours (*n* ≥ 4 per genotype per group). (**I**) Immunoblot analysis of Anapc13 protein in CD4^+^ T cells polarized under T_reg_ conditions shown in (H). The number on the top of the blot is the relative mean intensity of each Anapc13 band, and the right panel is the summary of the relative mean intensity. *P < 0.05 and **P < 0.01 (two-tailed Student’s *t* test).

We next determined mechanisms for Sf3b1-K700E–impaired T_reg_ differentiation and function. We first excluded the possibility that Foxp3 stability is affected, as the degradation rate of Foxp3 between *Foxp3^YFP-Cre^* and *Sf3b1^K700Efl/+^/Foxp3^YFP^* (fig. S5A) or between *Sf3b1^K700Efl/+^* and *Sf3b1^K700Efl/+^/CD4^Cre^* (fig. S5B) T_regs_ was equivalent. SF3B1 is a splicing factor, and Sf3b1-K700E mutation is known to affect cellular function by inducing aberrant splicing events to alter gene expression (*37*). Thus, we next performed deep RNA sequencing (RNA-seq) analysis to detect differential splicing events and transcriptomes between *Sf3b1^K700Efl/+^* and *Sf3b1^K700Efl/+^/CD4^Cre^* T_regs_. Since *Sf3b1^K700Efl/+^/CD4^-Cre^* cells displayed more marked defects in T_reg_ differentiation compared to *Sf3b1^K700Efl/+^/Foxp3^YFP-Cre^* cells (Fig. 1, E and F), *Sf3b1^K700Efl/+^/CD4^Cre^* cells were used in RNA-seq assay to capture the maximum differences in gene expression. The expression of the Sf3b1-K700E mutant was detected in all three RNA samples prepared from *Sf3b1^K700Efl/+^/CD4^Cre^* but not *Sf3b1^K700Efl/+^* T_regs_, confirming Cre-induced expression of Sf3b1-K700E (Fig. 5A). Consistent with the observed impaired T_reg_ differentiation of *Sf3b1^K700Efl/+^/CD4^Cre^* cells, *Foxp3*, *Id1*, *Irf4*, and *Myb*, which are known to positively regulate T_reg_ differentiation (*24*, *38*–*40*), were among the 30 down-regulated genes in these cells (Fig. 5, B and C) that were also confirmed by individual quantitative polymerase chain reaction (qPCR) analysis (Fig. 5D). However, forced expression of *Irf3*, *Myb*, or *Atf3* did not rescue T_reg_ differentiation in *Sf3b1^K700Efl/+^/CD4^Cre^* CD4^+^ T cells (fig. S5C). By alignment of genome DNA and coding sequence derived from RNA-seq, 2382 alternative splicing events in 1414 genes were identified. There were generally five different types of alternative splicing events, alternative 3′ splice site (A3SS), alternative 5′ splice site (A5SS), skipped exon, mutually exclusive exons, and retained intron (Fig. 5E). It is consistent with multiple reports that Sf3b1 mutants induce a high frequency of aberrant A3SS (*37*, *41*, *42*). Previous studies have identified a motif associated with canonical 3′ splice sites (3SSs) in cells expressing WT SF3B1 and a different motif associated with A3SS in cells expressing mutant SF3B1 (*37*, *41*–*43*). Our analysis of the sequence surrounding 3SS confirmed the motif associated with canonical 3SS in *Sf3b1^K700Efl/+^*cells expressing WT SF3B1 and a different motif associated with A3SS in *Sf3b1^K700Efl/+^/CD4^Cre^* cells expressing the Sf3b1-K700E mutant (Fig. 5F). These results indicate that Sf3b1-K700E induces aberrant splicing events in T_regs_. We next cross-examined 61 differentially expressed genes (Fig. 5B) and 1414 alternatively spliced genes to determine which differentially expressed genes are alternatively spliced and identified anaphase-promoting complex subunit 13 (*Anapc13*) (Fig. 5G). *Anapc13* was among the down-regulated genes in *Sf3b1^K700Efl/+^/CD4^Cre^* T_regs_ (Fig. 5C), which was confirmed by individual qPCR analysis (Fig. 5H). Anapc13 protein levels were also down-regulated in *Sf3b1^K700Efl/+^/CD4^Cre^* T_regs_ (Fig. 5I). *Anapc13* is known to regulate cell proliferation as deletion of this gene stalls cell cycle progression (*44*). *Sf3b1^K700Efl/+^/CD4^Cre^* T_regs_, which expressed lower levels of Anapc13 (Fig. 5, H and I), displayed reduced proliferation compared to the control *Sf3b1^K700Efl/+^* cells (Fig. 1H). Therefore, our RNA-seq analysis indicates that Sf3b1-K700E induces aberrant splicing events including the *Anapc*13 gene that is known to promote cell cycle progression but is down-regulated in slower proliferative *Sf3b1^K700Efl/+^/CD4^Cre^* T_regs_.

### Sf3b1-K700E–induced aberrant splicing event down-regulates *Anapc13* gene critical for T_reg_ differentiation

**Fig. 6.**
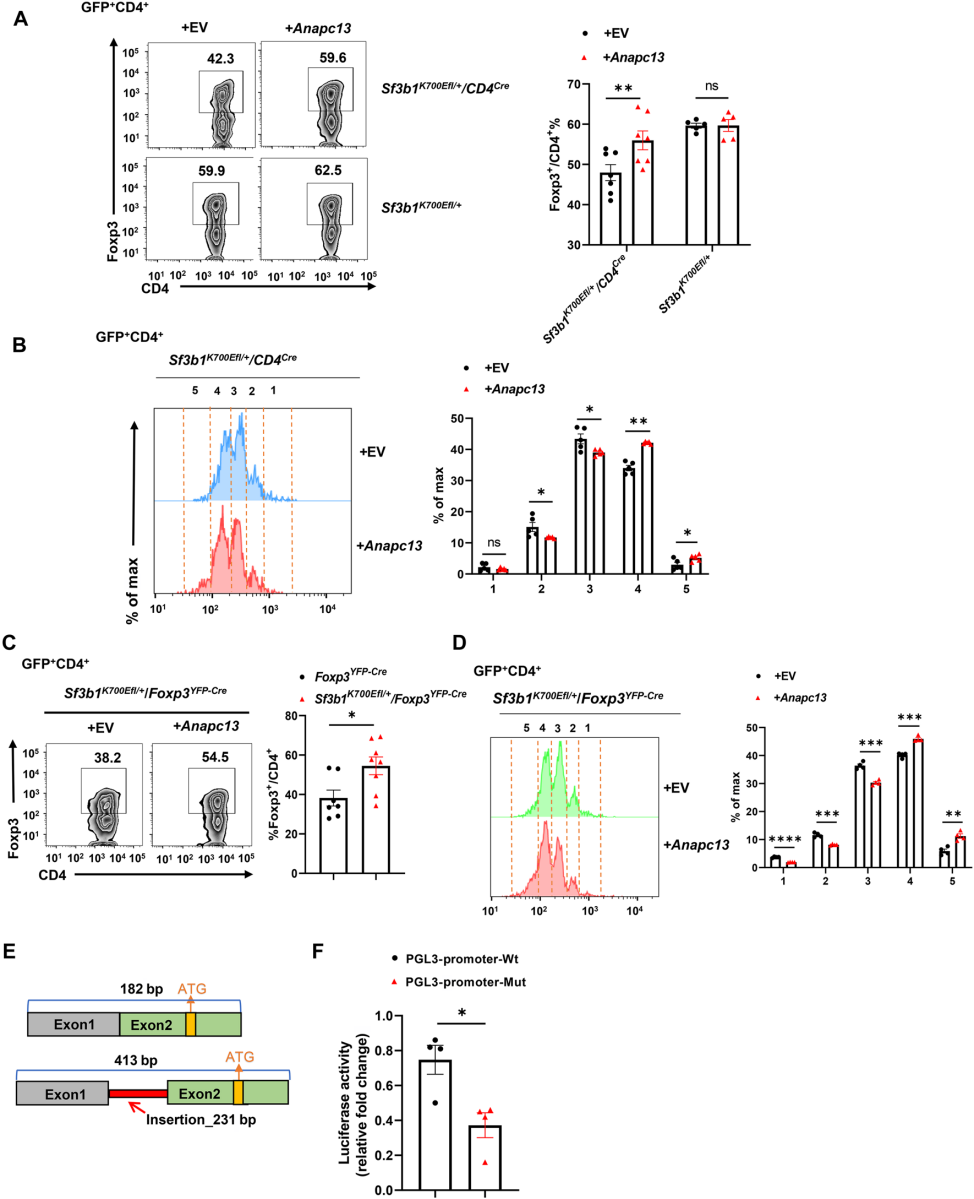
Sf3b1-K700E–induced aberrant splicing event down-regulates *Anapc13* gene critical for T_reg_ differentiation. (**A**) Representative flow cytometric analysis (left) and the percentage (right) of Foxp3^+^CD4^+^ T_reg_ among indicated genotypes of CD4^+^ T cells transduced with retrovirus expressing green fluorescent protein (GFP) ± *Anapc13* and polarized for 48 hours under T_reg_ conditions (*n* ≥ 4 per genotype per group). (**B**) Representative flow cytometric analysis (left) and the percentage (right) of the proliferative dye–labeled cells in the indicated peak of *Sf3b1^K700Efl/+^/CD4^Cre^* CD4^+^ T cells expressing GFP ± *Anapc13* and polarized under T_reg_ conditions shown in (A). (**C**) Representative flow cytometric analysis (left) and the percentage (right) of Foxp3^+^CD4^+^ T_reg_ among *Sf3b1^K700Efl/+^/Foxp3^YFP-Cre^* CD4^+^ T cells transduced with retrovirus expressing GFP ± *Anapc13* and polarized for 48 hours under T_reg_ conditions (*n* ≥ 4 per genotype per group). (**D**) Representative flow cytometric analysis (left) and the percentage (right) of proliferative dye–labeled *Sf3b1^K700Efl/+^/Foxp3^YFP-Cre^* CD4^+^ T cells indicated peak, expressing GFP ± *Anapc13* and polarized under T_reg_ conditions shown in C (*n* ≥ 4 per genotype). (**E**) Schematic representation of the WT (top) and aberrantly spliced *Anapc13* isoform. (**F**) Relative luciferase activity from the indicated reporter transfected into 293 T cells (*n* = 4 per genotype). EV, empty vector. Boxed region: cell population of interest. Data are from three experiments [(A) and (H); (B) to (E), right panels, presented as means ± SEM] or are from one representative of three independent experiments [(B) to (E), left panels]. *P < 0.05, **P < 0.01, ***P < 0.001, and ****P < 0.0005 (two-tailed Student’s *t* test).

To determine whether lower levels of Anapc13 are responsible for the impaired T_reg_ differentiation observed in *Sf3b1^K700Efl/+^/CD4^Cre^* CD4^+^ T cells, the effects of retrovirus-mediated expression of *Anapc13* on T_reg_ differentiation were determined. CD4^+^ T cells were labeled with dye for monitoring the proliferation of CellTrace Violet (CTV; fig. S6A). Forced expression of *Anapc13* stimulated T_reg_ differentiation (Fig. 6A) and cell proliferation (Fig. 6B) in *Sf3b1^K700Efl/+^/CD4^Cre^* but not *Sf3b1^K700Efl/+^* CD4^+^ T cells (fig. S6B). Similar stimulation of T_reg_ differentiation (Fig. 6C) and proliferation (Fig. 6D) by forced expression of *Anapc13* was also observed in *Sf3b1^K700Efl/+^/Foxp3^YFP-Cre^* cells, strongly supporting that the Sf3b1-K700E mutant decreases *Anapc13* expression, which then impairs T_reg_ differentiation by reducing cell proliferation.

To determine how the Sf3b1-K700E–induced aberrant splicing event affects *Anapc*13 gene expression, we designed primers to monitor the alternative splicing event based on our RNA-seq data. There were no alternative splicing events detected in naive CD4^+^ T cells from *Sf3b1^K700Efl/+^/CD4^Cre^* and *Sf3b1^K700Efl/+^* mice (fig. S6D, top). However, T_reg_ differentiation induced the alternative splicing event, indicated by the appearance of a high–molecular weight band, in *Sf3b1^K700Efl/+^/CD4^Cre^* cells expressing the Sf3b1-K700E mutant but not *Sf3b1^K700Efl/+^* cells that does not express the Sf3b1-K700E mutant (fig. S6D, middle). The alternative splicing event was not detected in purified T_regs_ from spleens before or after stimulation (fig. S6E), which correlates with no proliferation defects of stimulated purified T_regs_ (fig. S1I). We then performed sequence analysis of the WT and alternatively spliced bands and confirmed that alternative usage of 5SS results in an insertion of a 231–base pair (bp) fragment between exon 1 and exon 2 (fig. S6C). Since the start codon ATG is located in exon 2 (Fig. 6E), exon 1 and the inserted 231-bp fragment are in the 5′UTR. To determine whether the inserted fragment affects gene expression, a WT DNA fragment containing exon 1 and exon 2 with or without alternative spliced 231-bp DNA fragment (Fig. 6E) was cloned between the SV40 promoter and a luciferase reporter gene. Insertion of 231 bp substantially inhibited luciferase activity (Fig. 6F). Deletion of a portion (about 80 bp) from the 231-bp insertion reduced inhibitory effects on luciferase activity (fig. S6F). Therefore, the Sf3b1-K700E–induced aberrant splicing event leads to the insertion of the 231-bp fragment that inhibits the expression of *Anapc13* critical for T_reg_ differentiation via controlling cell proliferation.

### Forced expression of *Anapc13* restores the function of *Sf3b1^K700Efl/+^/CD4^Cre^* T_regs_ in the prevention of colitis

**Fig. 7.**
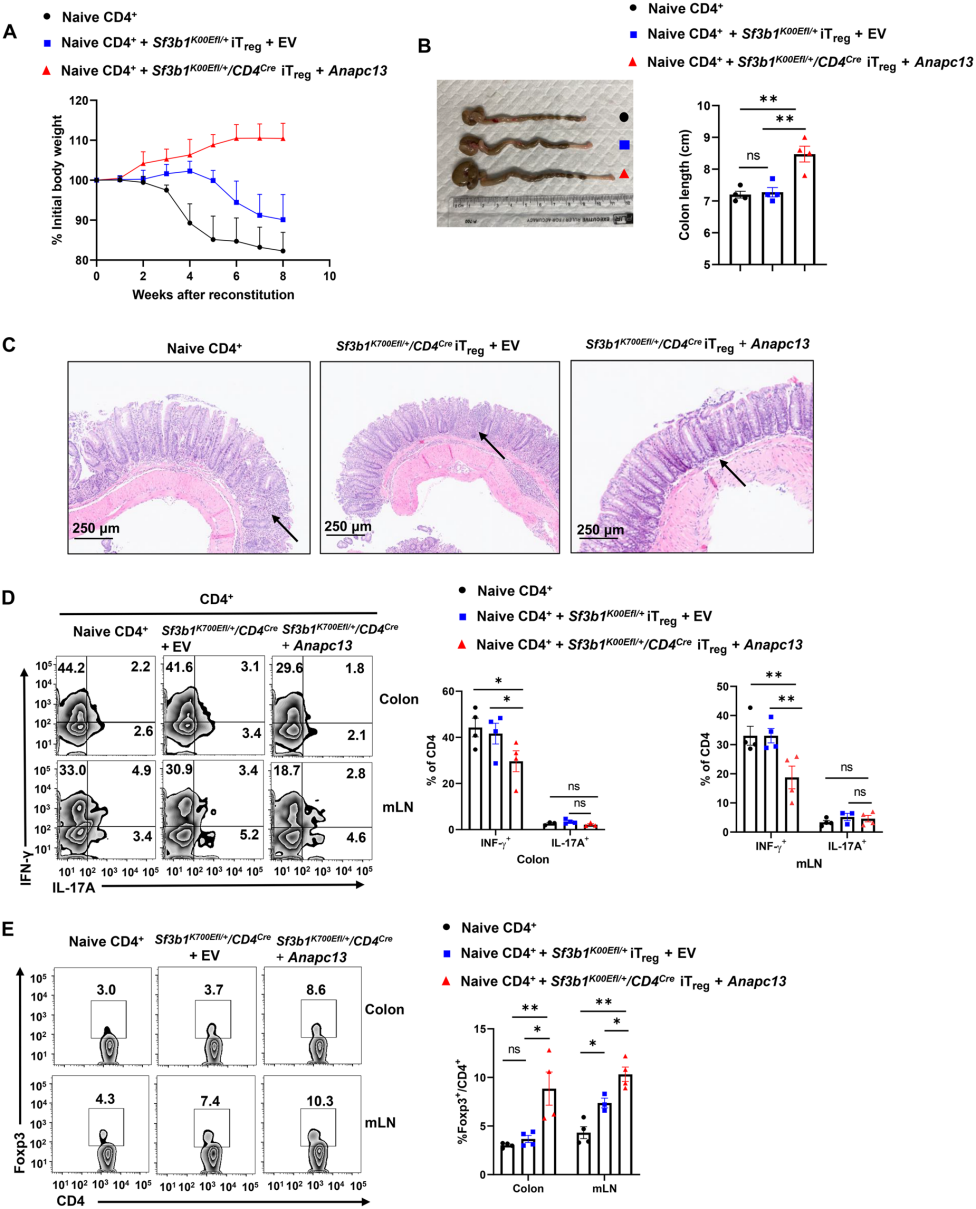
Forced expression of *Anapc13* rescues T_reg_ differentiation and function. (**A**) Body weight of *Rag1*^−/−^ recipients over time after adoptive transfer of WT CD45RB^hi^CD25^−^CD4^+^ naive T cells alone or in combination with differentiated iT_regs_ retrovirally expressing GFP (EV) along or together with *Anapc13* (+*Anapc13*). (**B**) Representative image of colons (left) and colon length (right) of colitis-induced recipient shown in (A) (*n* ≥ 4 per genotype). (**C**) H&E-stained colon sections from colitis-induced recipients shown in (A). (**D**) Representative flow cytometric analysis (left) and percentage (right) of CD4^+^IL-17A^+^ and CD4^+^IFN-γ^+^ cells recovered from colons of colitis-induced recipients shown in (A) (*n* ≥ 4 per group). (**E**) Representative flow cytometric analysis (left) and percentage (right) of Foxp3^+^CD4^+^ T_regs_ recovered from colon, spleen, and mLN of colitis-induced recipients shown in (A) (*n* ≥ 4 per group). Boxed area: cell population of interest. Data are from three experiments [(A); (B), (D), and (E), right panels, presented as means ± SEM] or are from one representative of three independent experiments [(C); (B), (D), and (E), left panels]. *P < 0.05 and **P < 0.01 (two-tailed Student’s *t* test).

To determine whether reduced *Anapc13* is responsible for the impaired function of *Sf3b1^K700Efl/+^/CD4^Cre^* T_regs_ in the prevention of colitis, an adoptive transfer colitis model was used to determine the function of *Anapc13* in the impaired function of *Sf3b1^K700Efl/+^/CD4^Cre^* T_regs_. Naive CD4^+^ T cells from *Sf3b1^K700Efl/+^/CD4^Cre^* mice were transduced with retrovirus expressing green fluorescent protein (GFP; +EV) along or together with *Anapc13* (+*Anapc13*) and differentiated into T_regs_, followed by adoptive transfer of T_reg_ differentiated GFP^+^ cells to *Rag1*^−/−^ mice to inhibit colitis induced by adoptively transferred naive CD4^+^ T cells (fig. S7A). Overexpression of *Anapc13* is confirmed 48 hours after viral infection (fig. S7B). Although severe colitis was induced by adoptive transfer of naive CD4^+^ along, the colitis was greatly inhibited by cotransfer of *Sf3b1^K700Efl/+^/CD4^Cre^* T_regs_ expressing *Anapc13* but not GFP alone, indicated by the prevention of weight loss (Fig. 7A), shortening colon (Fig. 7B), tissue damages (Fig. 7C), and reduced IFN-γ but not IL-17 production from CD4^+^ T cells [see Fig. 7D and fig. S7 (C and D) for gating strategy] together with increased T_regs_ detected in colon and lymph nodes [see Fig. 7E and fig. S7 (C and D) for gating strategy]. Therefore, Sf3b1-K700E impairs T_reg_ function via induction of an aberrant splicing event to inhibit *Anapc13* expression.

### AML grows faster in aged *Sf3b1^K700Efl/+^/Foxp3^YFP-Cre^* mice

**Fig. 8.**
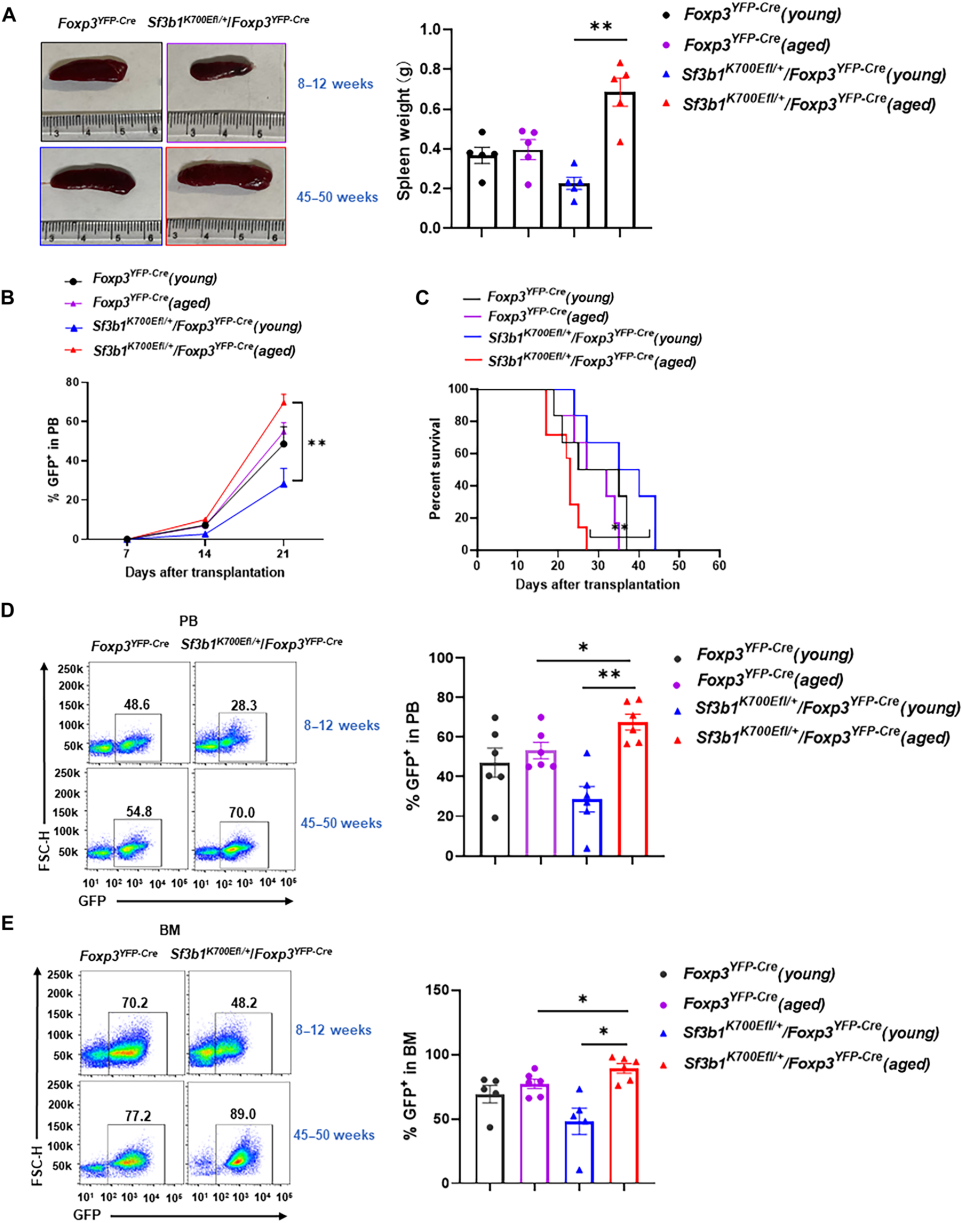
AML grows faster in aged *Sf3b1^K700Efl/+^/Foxp3^YFP-Cre^* mice. (**A**) Representative image (left) and weight (right) of the spleens from indicated mice implanted with AML. (**B**) The percentage of GFP^+^ AML cells in peripheral blood (PB) of indicated mice at different times after implantation of AML (*n* = 7 per genotype per group). (**C**) Kaplan-Meier curves showing the survival of the indicated mice implanted with AML (*n* = 7 per genotype per group). (**D** and **E**) Representative flow cytometric analysis (left) and percentage (right) of GFP^+^ AML cells in the PB (E) and bone marrow [BM, (F)] from indicated mice 21 days after AML implantation. Boxed area: cell population of interest. Data are from three experiments [(A) and (B); (C) to (E), right panels, presented as means ± SEM] or are from one representative of three independent experiments [(C) to (E), left panels]. The statistical significance for (B) was determined using the log-rank (Mantel-Cox) test. *P < 0.05 and **P < 0.01 (two-tailed Student’s *t* test).

To determine whether T_reg_-specific expression of Sf3b1-K700E affects cancer development, we compared the growth of AML (acute myeloid leukemia) in *Foxp3^YFP-Cre^* and *Sf3b1^K700Efl/+^/Foxp3^YFP-Cre^* mice (fig. S8A). Sf3b1-K700E somatic mutation in AML was reported (*45*) and also identified by public cohort mining of DNA sequence from The Cancer Genome Atlas program (fig. S8B). We first noticed that aged *Sf3b1^K700Efl/+^/Foxp3^YFP-Cre^* mice had much larger spleen size and weight than aged WT *Foxp3^YFP-Cre^* control mice (Fig. 8A). On the other hand, young *Sf3b1^K700Efl/+^/Foxp3^YFP-Cre^* mice had smaller spleen size and weight compared to young WT *Foxp3^YFP-Cre^* control mice. Transplanted AML cells (MLL-AF9-GFP) grew faster in aged *Sf3b1^K700Efl/+^/Foxp3^YFP-Cre^* mice compared to aged *Foxp3^YFP-Cre^* control mice (Fig. 8B). In contrast, AML grew rather slower in young *Sf3b1^K700Efl/+^/Foxp3^YFP-Cre^* mice compared to young *Foxp3^YFP-Cre^* control mice. Consistently, the survival curves showed that more aged *Sf3b1^K700Efl/+^/Foxp3^YFP-Cre^* mice died of AML compared to aged *Foxp3^YFP-Cre^* mice, whereas younger *Sf3b1^K700Efl/+^/Foxp3^YFP-Cre^* mice survived better compared to young *Foxp3^YFP-Cre^* mice (Fig. 8C). Analysis of GFP^+^ AML cells in peripheral blood (PB; see Fig. 8D and fig. S8C for gating strategy) and bone marrow (BM; see Fig. 8E and fig. S8D for gating strategy) confirmed that aged *Sf3b1^K700Efl/+^/Foxp3^YFP-Cre^* mice had higher percentage of AML cells compared to aged *Foxp3^YFP-Cre^* mice. On the other hand, young *Sf3b1^K700Efl/+^/Foxp3^YFP-Cre^* mice had a lower percentage of AML cells compared to young *Foxp3^YFP-Cre^* mice. Therefore, our results demonstrate that SF3B1-K700E mutation in T_regs_ promotes AML growth in aged but not young mice. The faster growth of AML in aged *Sf3b1^K700Efl/+^/Foxp3^YFP-Cre^* mice correlates with observed more inflammation (Fig. 4), whereas the slower AML growth in young *Sf3b1^K700Efl/+^/Foxp3^YFP-Cre^* mice correlates with less inflammation (Fig. 4), indicating the link between AML growth and inflammation.

## DISCUSSION

Hotspot heterozygous point mutations in SF3B1 are the most common across cancer types including CLL (*46*, *47*), MDS (*4*, *13*), and uveal melanoma (*48*–*50*). Previous research focuses on understanding how SF3B1 mutations, particularly the highly frequent SF3B1-K700E mutation, promote tumorigenesis (*14*–*16*, *51*). SF3B1 mutations are identified in hematopoietic progenitors that differentiate into immune cells including T_regs_. Here, we demonstrated that T_reg_-specific expression of SF3B1-K700E broke immune tolerance, resulting in autoinflammation. Mechanically, we showed that SF3B1-K700E induced an aberrant splicing event at the *Anapc13* gene, which led to an insertion of a 231-bp fragment to the 5′UTR region. This insertion inhibited the expression of *Anapc13* which is required for the proliferation of T_regs_.

Anapc13 is a critical subunit of anaphase-promoting complexes (APC) which regulates cell cycle progression by controlling the degradation of cell cycle regulators including securing and cyclin B (*52*). APC has ubiquitin ligase activity and thus regulates the degradation of cell cycle regulators via ubiquitination-dependent pathways. Sequential degradation of different cell cycle regulators is a critical mechanism for controlling cell cycle progression. Inhibition of APC-dependent degradation prevents cell cycle progression. Anapc13 is a less studied protein compared to the other subunits of APC complexes. Anapc13 is important for stabilizing the APC complexes and is required for the ubiquitin ligase activity of APC. Deletion of *Anapc13* stalls cell cycle progression (*44*). Consistent with the critical function of Anapc13 in the regulation of cell cycle progression, *Sf3b1^K700Efl/+^/CD4^Cre^* T_regs_ that express lower levels of Anapc13 display reduced proliferation. Reduced proliferation is responsible for impaired *Sf3b1^K700Efl/+^/CD4^Cre^* T_reg_ differentiation, as forced expression of Anapc13 restores proliferation and T_reg_ differentiation. Furthermore, forced expression of Anapc13 in *Sf3b1^K700Efl/+^/CD4^Cre^* T_regs_ restores its function in preventing colitis in vivo. Therefore, we demonstrated that SF3B1-K700E regulates T_reg_ differentiation via regulating *Anapc13*-dependent cell cycle progression. SF3B1-K700E has been shown to regulate tumor cell proliferation via other mechanisms (*16*, *53*). Therefore, how SF3B1 mutants affect cellular function is cell type–dependent, which is likely due to differential alternative splicing events induced by SF3B1-K700E in different cell types. Our study shows that SF3B1 induces a 3′ alternative splicing event at the *Anapc13* gene in T_regs_, which results in an insertion of a 231-bp fragment at 5′UTR. Furthermore, we show that insertion of the 231-bp fragment inhibits *Anapc13* transcription. Insertion of the 231-bp fragment in front of the luciferase gene also inhibits luciferase activity, suggesting that this inhibition mechanism may not be gene-specific. It is thus worth investigating how the insertion of this fragment inhibits gene expression in the future.

T_regs_ are a double-edged sword for tumor development. On one hand, the tumor microenvironment, particularly solid tumors, is enriched with immunosuppressive cells including T_regs_ that allow tumor cells to escape from immune surveillance and prevent antitumor immune responses (*54*). On the other hand, inflammation resulting from impaired T_reg_ differentiation and function has been shown to fuel tumor progression and metastasis (*55*–*58*). We showed that T_reg_-specific expression of SF3B1-K700E generates an inflammatory environment, indicated by pro-inflammatory cytokine production and lymphocyte infiltration. It remains unknown about the relationship between our observed SF3B1-K700E–induced inflammation and tumor development. Large-scale cancer genome sequencing projects have identified recurrent somatic mutations in splicing factors in several types of hematological malignancies including MDS and AML (*4*–*6*). Patients with MDS and AML have been found to produce a variety of inflammatory cytokines (*59*–*63*), and these inflammatory mediators have been shown to directly support the growth of stem cells containing SF3B1 mutation, resulting in the development of animal models of MDS (*59*, *64*–*67*). Our results show that AML grows faster in aged, but not young, mice expressing SF3B1-K700E in T_regs_. The increased AML growth is associated with heightened inflammation observed in aged mice. Cancer and inflammation also dominate in aged humans (*59*, *68*–*70*), which shows the link between cancer and inflammation. However, it remains to be determined how the inflammation resulting from SF3B1-K700E–impaired T_reg_ function promotes AML development.

Splicing factor mutations have been associated with different types of cancers including both hematological malignancies and solid tumors (*71*). However, so far, most studies focus on revealing how splicing factor mutations intrinsically promote oncogenesis. Little is known about how the splicing factor mutations affect the function of other somatic tissues. Our study demonstrates that splicing factor mutations could substantially interfere with other cell functions, immune cells in this study in addition to tumor cells. Furthermore, splicing factor mutations that affect the function of other tissues could contribute to tumor development. Understanding these extrinsic effects of splicing factor mutations will facilitate the development of effective therapies for the treatment of splicing factor mutation-associated cancers in general.

## MATERIALS AND METHODS

### Mice

Transgenic *CD4^Cre^* (*TgCd4^cre^*, 022071), *Rag1*^−/−^ (*Rag1^tm1Mom^*, 002216), and *C57BL* (*B6*, 000664) mice were purchased from the Jackson Laboratory. *Sf3b1^K700E/fl^* mice were obtained from L.W. Laboratory (Systems Biology-BRI, Beckman Research Institute, City of Hope, CA). *OT-II* mice were obtained from J. Yu Laboratory (Department of Hematology and Hematopoietic Cell Transplantation, City of Hope, CA), and *Foxp3^YFP-Cre^* mice were obtained from M. Boldin Laboratory (Molecular and Cellular Biology, Beckman Research Institute, City of Hope, CA). All mice were bred into the C57BL/6J background and housed under specific pathogen–free conditions in the Animal Resource Center at the Beckman Research Institute of City of Hope under protocols approved by the Institutional Animal Care and Use Committee (IACUC; #07023). Mice were 10 to 12 weeks of age for EAE studies and 8 to 10 weeks of age for other experiments, unless indicated otherwise, with littermates age- and sex-matched across experimental groups.

### Antibodies and cytokines

Monoclonal antibodies against mouse CD3 (145-2C11), CD28 (37.51), IL-4 (11B11), and IFN-γ (XMG1.2), as well as phycoerythrin (PE)-conjugated anti-CD8 (dilution ratio, 1:100; 53-6.7), allophycocyanin-conjugated anti-Foxp3 (dilution ratio, 1:100; FJK-16s), PE-indotricarbocyanine (Cy7)–conjugated anti–IL-17A (dilution ratio, 1:100; eBio17B7), APC-conjugated anti–IFN-γ (dilution ratio, 1:100; XMG1.2), and Live/Dead Fixable Near-IR Dead Cell Stain (dilution ratio, 1:1000; L34976) were from Invitrogen. PE-conjugated anti-CD25 (dilution ratio, 1:100; PC61), Brilliant Violet (BV) 605–conjugated anti-CD4 (dilution ratio, 1:100; RM4-5), BV 421–conjugated anti-CD3 (dilution ratio, 1:100; 145-2C11), APC-conjugated anti-CD45 (dilution ratio, 1:100; I3/2.3), PE-Cy7–conjugated anti-CD45RB (dilution ratio, 1:100; C363-16A), PE-Cy7–conjugated anti-CD62L (dilution ratio, 1:100; MEL-14), APC-Cy7–conjugated anti-CD44 (dilution ratio, 1:100; IM7), PE-conjugated anti-CD73 (dilution ratio, 1:100; TY/11.8), PE-Cy7–conjugated anti-CD39 (dilution ratio, 1:100; Duha59), APC-Cy7–conjugated anti-CD45 (dilution ratio, 1:100; 30-F11), PE-Cy7–conjugated anti–CTLA-4 (dilution ratio, 1:100; UC10-4B9), and recombinant murine IL-2 were from BioLegend. APC-conjugated anti-CD25 (dilution ratio, 1:100; PC61) was from BD Biosciences. Antibodies against Anapc13 (dilution ratio, 1:1000; PA5-20956, Thermo Fisher Scientific) and β-actin (dilution ratio, 1:1000; 4970L, Cell Signaling) were used for immunoblotting analysis. Recombinant mouse TGF-β was from Miltenyi Biotec.

### Plasmids

The retroviral vector murine stem cell virus (MSCV)–internal ribosomal entry site (IRES)–GFP was a gift from W. S. Pear (University of Pennsylvania). cDNA encoding *Anapc13* and *ATF3* was cloned into MSCV-IRES-GFP vector. IRF4-MIEG-GFP was a gift from M. H. Kaplan Laboratory (Indiana University School of Medicine); 7×E-Box:Renilla (plasmid #124532) and MSCV-PIG-Myb (plasmid #66988) were purchased from Addgene. pGL3-promoter (plasmid #E1761) was purchased from Promega (Madison, WI).

### Flow cytometry

For surface staining, cells isolated from mice or in vitro culture were directly stained with antibodies and/or fixable live/dead dye with 2% fetal bovine serum (FBS) and 1 mM EDTA at 4°C for 15 min. For transcription factor staining, cells prestained with surface markers were fixed and permeabilized in TF Fix/Perm buffer (BD Biosciences) at 4°C for 20 min, washed once with TF Perm/Wash buffer, and stained with target markers in the TF Perm/Wash buffer at 4°C for 15 min. For intracellular cytokine analysis, cells were stimulated with phorbol 12-myristate 13-acetate (50 ng/ml; Sigma-Aldrich) and ionomycin (750 ng/ml; Sigma-Aldrich) at 37°C for 3 hours in the presence of GolgiStop (BD Biosciences) before staining. After stimulation, cells were stained with surface markers and then fixed and permeabilized with Cytofix/Cytoperm buffer (BD Biosciences) for 20 min followed by staining cytokines in the Perm/Wash buffer (BD Biosciences) after washing. The expression of surface and intracellular markers was analyzed with a BD LSRFortessa flow cytometer.

### Isolation of naive CD4^+^ T cells and in vitro T_reg_ differentiation

Naive CD4^+^ T cells were isolated from mouse spleens by negative selection using the Naive CD4^+^ T Cell Isolation Kit (Miltenyi Biotec). Suspensions of 3 × 10^5^ cells per well of RPMI 1640 medium (Corning Inc.) containing 2 mM l-glutamine, 50 μM β-mercaptoethanol, penicillin (100 U/ml), streptomycin (100 mg/ml), and 10% FBS (Corning Inc.) were cultured in 48-well plates precoated with rabbit anti-hamster (0.1 mg/ml). The medium was supplemented with hamster anti-CD3 (0.25 μg/ml), hamster anti-CD28 (1 μg/ml), TGF-β (5 ng/ml), anti–IL-4 (2.5 μg/ml), and anti–IFN-γ (2.5 μg/ml) for T_reg_ differentiation for up to 48 hours.

### In vivo induction of iT_regs_ by adoptively transferring naive CD4^+^ T cells

Splenic cells were collected from *Foxp3^YFP-Cre^* or *Sf3b1^K700E/fl^/Foxp3^YFP-Cre^* mice (8 to 10 weeks). Naive CD4^+^ T cells were first enriched by negative selection using the Naive CD4^+^ T Cell Isolation Kit, and then CD4^+^YFP^−^ cells were sorted via FACSAria Fusion (BD Biosciences) to enable a high purity of ≥99.0%. A total of 4 × 10^5^ naive CD4^+^ T cells were intraperitoneally injected into sex-matched *Rag1*^−/−^ mice. Three weeks after adoptive transfer, cells from the spleen and mLN of *Rag1*^−/−^-recipient mice were collected and analyzed.

### In vivo induction of iT_regs_ by oral tolerance

Splenic cells were collected from *OT-II/ Sf3b1^K700E/fl^* or *OT-II/ Sf3b1^K700E/fl^ /CD4^Cre^* mice (8 to 10 weeks), naive CD4^+^ T cells were first enriched by negative selection using the Naive CD4^+^ T Cell Isolation Kit, and CD4^+^CD25^−^ cells were then sorted via FACSAria Fusion to enable a high purity of ≥99.0%. A total of 3 × 10^6^ cells were intraperitoneally injected into sex-matched *Rag1*^−/−^ mice. After 24 hours, recipient mice were provided with grade VI OVA (20 mg/ml; Sigma-Aldrich) ad libitum in drinking water for 5 days. Drinking water containing OVA was changed every 2 days. Cells were collected from the colon, spleen, and mLN on day 6 for analysis.

### Induction and assessment of EAE

EAE was induced and assessed according to the manufacturer’s instructions (Hooke Laboratories, Lawrence, MA). Briefly, *Foxp3^YFP-Cre^* or *Sf3b1^K700E/fl^ /Foxp3^YFP-Cre^* mice were immunized with 200 mg of MOG35-55 (Hooke Laboratories) in complete Freund’s adjuvant by subcutaneous injection at two dorsal sites of mice, followed by two intraperitoneal injections of 80 ng of pertussis toxin at days 0 and 1. The severity of EAE was monitored and evaluated on a scale from 0 to 5 according to Hooke Laboratories’ guidelines. Briefly, 0 represents no disease, 1 represents a paralyzed tail, 2 represents hindlimb weakness, 3 represents hindlimb paralysis, 4 represents hindlimb and forelimb paralysis, and 5 represents moribund and death. When a mouse was euthanized because of severe paralysis, a score of 5 was entered for that mouse for the rest of the experiment.

### In vivo T_reg_ suppression assay

Colitis was induced in sex-matched *Rag1*^−/−^ mice by intraperitoneally injecting 4 × 10^5^ CD45RB^hi^CD25^−^CD4^+^ naive T cells sorted from the spleen of C57BL mice (8 to 10 weeks). For nT_reg_ suppression assay in *Sf3b1^K700E/fl^ /Foxp3^YFP-Cre^* strain mice, 2 × 10^5^ CD4^+^YFP^+^ T_regs_ sorted from the spleen of 8- to10-week-old *Foxp3^YFP-Cre^* or *Sf3b1^K700E/fl^ /Foxp3^YFP-Cre^* mice were mixed with 4 × 10^5^ CD45RB^hi^CD25^−^CD4^+^ naive T cells from C57BL mice and injected into sex-matched *Rag1*^−/−^ mice.). For nT_reg_ suppression assay in *Sf3b1^K700E/fl^ /CD4^Cre^* strain mice, 2 × 10^5^ CD4^+^CD25^+^ T_regs_ sorted from the spleen of 8- to 10-week-old *Sf3b1^K700E/fl^* or *Sf3b1^K700E/fl^/CD4^Cre^* mice were mixed with 4 × 10^5^ CD45RB^hi^CD25^−^CD4^+^ naive T cells from C57BL mice and injected into sex-matched *Rag1*^−/−^ mice. For the iT_reg_ suppression assay, naive CD4^+^ T cells from *Sf3b1^K700E/fl^ /CD4^Cre^* mice were in vitro activated as above and retrovirally transducted with EV or *Anapc13* alone. After differentiation for 3 days, 2 × 10^5^ iT_regs_ transducted with EV or *Anapc13* alone were mixed with 4 × 10^5^ CD45RB^hi^CD25^−^CD4^+^ naive T cells from C57BL mice and injected into sex-matched *Rag1*^−/−^ mice as above. Mice were weighed immediately following T cell transfer and weekly thereafter. Eight to 9 weeks after cell transfer, the colon, spleen, and mLN were removed from *Rag1*^−/−^-recipient mice for analysis.

### In vitro T_reg_ suppression assay

Sorted CD4^+^CD25^−^ T cells were labeled with CTV (dilution ratio, 1:1000; C34557, Invitrogen) and served as responder CD4^+^ T (T_resp_) cells. T_resp_ cells (6 × 10^5^ cells/ml) were cocultured with CD4^+^YFP^+^ T_regs_ sorted from the spleens of *Foxp3^YFP-Cre^* or *Sf3b1^K700E/fl^ /Foxp3^YFP-Cre^* mice in 48-well plates [precoated with rabbit anti-hamster (0.1 mg/ml)] in culture medium supplemented with hamster anti-CD3 (0.25 μg/ml) and hamster anti-CD28 (1 μg/ml) for 3 days. The ratios of T_resp_ cells to T_regs_ were 1:0, 1:1, and 2:1 for T_regs_ sorted from mice and 1:0, 1:1, and 2:1 for T_regs_ sorted from in vitro differentiation. The proliferation of T_resp_ cells was assessed by flow cytometry.

### Histology study

Tissues were cleaned and fixed with 4% paraformaldehyde, embedded in paraffin, and then sectioned and stained with hematoxylin and eosin.

### RNA-seq and analysis

Naive CD4^+^ T cells isolated from *Sf3b1^K700E/fl^* or *Sf3b1^K700E/fl^ /Cd4^Cre^* mice were differentiated into T_regs_ in 24-well plates in the presence of TGF-β (5 ng/ml), anti–IL-4, and anti–IFN-γ for 36 hours. CD4^+^ T cells after 36 hours of T_reg_ differentiation were collected and subjected to RNA extraction with the RNeasy Mini Kit (QIAGEN). Each group has three replicates from different mice. Quality control, library preparation, and sequencing were performed at Novogene. The analysis was performed through Partek Flow. Briefly, the sequence reads were aligned to the mouse whole genome (GRCm38) with validation of quality through pre-alignment and post-alignment quality assurance/quality control. Aligned reads were further subjected to quantification using the Partek E/M algorithm and normalization to counts per million with 0.001 added to each. The identification of differentially expressed features was performed through the Partek GSA algorithm that applies multiple statistical models to each gene. Genes with total counts of more than 10 were considered to be statistically expressed in the cells. The expression values of pathogenic genes were extracted and subjected to ingenuity pathway analysis and network analysis.

### Differential alternative splicing analyses

Alternative splicing analysis was performed using rMATS (version 4.0.2) (*72*), a Python algorithm used to identify alternative splicing events by quantifying exon-exon junction spanning reads on annotated splice junctions in rat GENCODE Rnor_6.0 assembly. Differentially spliced mRNAs were defined as a false discovery rate of <0.05 and a minimum inclusion level difference of >10% or <−10%. Three mutant Sf3b1^K700E^ replicates and three WT replicates were compared.

### Reverse transcription quantitative real-time PCR

Total RNA of cells was extracted according to the manufacturer’s guidelines using the RNeasy Mini Kit (QIAGEN). The first-strand cDNA synthesis was performed by reverse transcription using a Tetro cDNA Synthesis Kit (Bioline). Subsequent qPCR was performed using PowerUp SYBR Green Master Mix (Applied Biosystems) in the QuantStudio 3 Real-Time PCR System (Thermo Fisher Scientific). The primers used for qPCR and reverse transcription PCR are listed in table S1. The amplification efficiency of all primers has been tested, and the optimized conditions were used in all qPCRs. Gene expression was calculated with the ΔΔCt method normalized to the control gene encoding β-actin, and all measurements were performed in triplicate.

### Retroviral transduction

Vectors were firstly transfected to Platinum-E (Plat-E; Cell Biolabs) retroviral packaging cells by using BioT transfection reagent (Bioland Scientific) followed by a changing fresh medium at 24 hours. The virus-containing medium collected at 48 and 72 hours was filtered with a 0.45-μm polyvinylidene difluoride (PVDF) syringe filter (Millipore), followed by either direct transduction to T cells or stored at −80°C for later use. Naive CD4^+^ T cells were labeled with Cell Trace Violet (dilution ratio, 1:1000; C34557, Invitrogen) and then activated by hamster anti-CD3 (0.25 μg/ml) and hamster anti-CD28 antibodies (1 μg/ml) in precoated plates for 20 hours before transduction. Transduction to activated CD4^+^ T cells was performed by spin infection with viral supernatants (2500 g, 30°C for 2 hours) in the presence of polybrene (10 μg/ml; Sigma-Aldrich). Afterward, the plates were kept in the incubator at 37°C for 3 hours. The viral supernatant was replaced by a fresh culture medium with polarizing cytokines and antibodies for T_reg_ differentiation.

### Western blotting

For Western blotting, cells were lysed in radioimmunoprecipitation assay buffer containing 20 mM tris-HCl (pH 7.4), 150 mM NaCl,1 mM Na_2_ EDTA, 1 mM EGTA, 1% NP-40, 1% sodium deoxycholate, 2.5 mM sodium pyrophosphate, 1 mM β-glycerophosphate, 1 mM Na_3_VO_4_, and leupeptin (1 μg/ml) on ice for 45 min and spun down at 15,000 rpm for 10 min at 4°C to collect the extract. The 2× Laemmli sample buffer (Bio-Rad) containing β-mercaptoethanol was mixed with cell extract and heated at 95°C for 5 min. Protein was separated by SDS–polyacrylamide gel electrophoresis and transferred to PVDF membrane (Millipore). Target proteins were sequentially immunoblotted with relevant primary antibodies and fluorescent secondary antibodies (LI-COR Biosciences) followed by measuring fluorescent intensity with LI-COR Odyssey blot imager (LI-COR Biosciences).

### Luciferase assay

The 5′UTR DNA sequence of *Anapc13* as the WT sequence and the 5′UTR DNA sequence including the insertion of the 231-bp DNA sequence of *Anapc13* as the Mut sequence. Both of the sequences were inserted between the promoter and luciferase of the basic PGL3 promoter vector. To measure the luciferase activity, the PGL3-promoter vector (1 μg), PGL3-promoter-WT, or PGL3-promoter-Mut luciferase vector alone (1 μg) was delivered to 4 × 10^5^ human embryonic kidney 293T cells seeded in a six-well plate. Renilla luciferase vector (200 ng) was cotransfected to cells in each group for normalizing different transfection efficiencies. An empty vector was used to adjust the total plasmid DNA to the same amount. Luciferase activity was measured in Dual-Luciferase Reporter Assay System (Promega, Madison, WI) per the manufacturer’s instruction at 1-day posttransfection in a Synergy HTX multi-mode reader (Agilent, Santa Clara, CA). Briefly, after background subtraction, Firefly luciferase activities were normalized to Renilla luciferase values, followed by an additional normalization of all Firefly to Renilla ratios to the PGL3-promoter group.

### AML mouse model

Murine MLL-AF9-GFP cell (C58BL/6) was a gift from Y.H. Kuo (City of Hope). MLL-AF9-GFP AML cells were transplanted (1 × 10^6^ cells) via tail vein intravenous (iv) injection into recipient mice to generate AML. Engraftment of GFP^+^ cells in PB was monitored by flow cytometry every week after transplantation. After 3 weeks, PB and BM cells were collected and assessed for AML burden by flow cytometry.

### Statistics and reproducibility

The results were analyzed for statistical significance with unpaired Student’s *t* test or one-way or two-way analysis of variance (ANOVA) where appropriate. The log-rank test was used to assess significant differences between survival curves. All data are presented as means ± SEM. *P* values are calculated using GraphPad Prism and presented where the statistical significance (*P* < 0.05) was found.
