# Supplementary material for: Cancer-associated SF3B1-K700E mutation controls immune responses by regulating Treg function via aberrant Anapc13 splicing
Source: Sci Adv. 2024 Sep 20;10(38):eado4274. doi: 10.1126/sciadv.ado4274 (PMC11414738; doi:10.1126/sciadv.ado4274)
Supplement: Supplementary file 1 — Figs. S1 to S8 Table S1 [file sciadv.ado4274_sm.pdf]

Science Advances
AAAS

Supplementary Materials for

Cancer-associated SF3B1-K700E mutation controls immune responses by regulating T_{reg} function via aberrant *Anapc13* splicing

Yun Shi *et al.*

Corresponding author: Zuoming Sun, zsun@coh.org

Sci. Adv. **10**, eado4274 (2024)
DOI: 10.1126/sciadv.ado4274

This PDF file includes:

Figs. S1 to S8
Table S1

Fig. S1

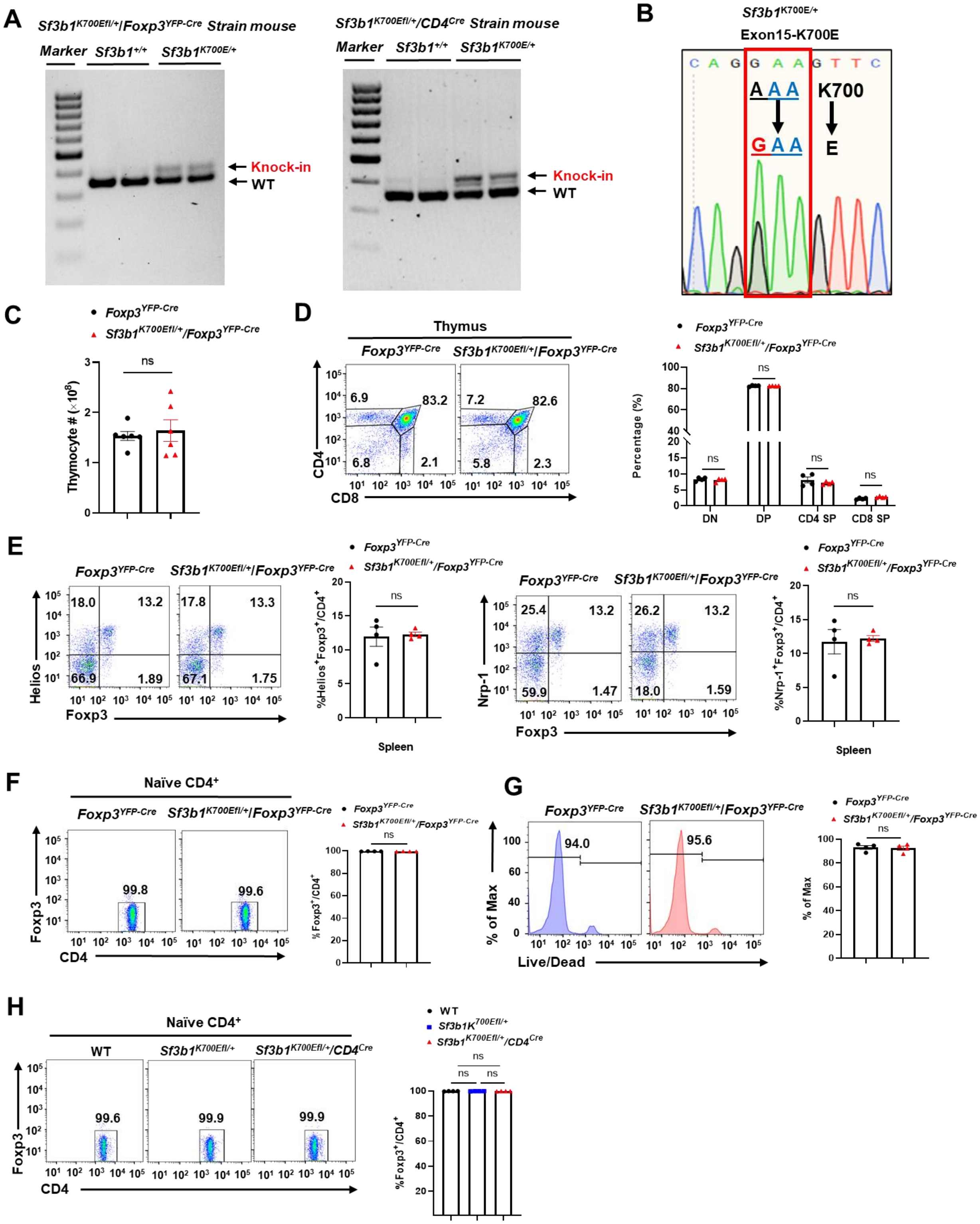
A
Sf3b1K700Efl/+/Foxp3YFP-Cre Strain mouse
Sf3b1K700Efl/+/CD4Cre Strain mouse
Marker
Sf3b1+/+
Sf3b1K700E/+
Knock-in
WT
B
Sf3b1K700E/+
Exon15-K700E
AAA
GAA
K700
E
C
Foxp3YFP-Cre
Sf3b1K700Efl/+/Foxp3YFP-Cre
Thymocyte # (×10⁸)
ns
D
Thymus
CD4
CD8
Percentage (%)
DN
DP
CD4 SP
CD8 SP
E
Helios
Foxp3
%Helios+Foxp3+/CD4+
Spleen
Nrp-1
%Nrp-1+Foxp3+/CD4+
F
Naïve CD4+
%Foxp3+/CD4+
G
% of Max
Live/Dead
H
WT
Sf3b1K700Efl/+
Sf3b1K700Efl/+/CD4Cre

Fig. S1-continued

I

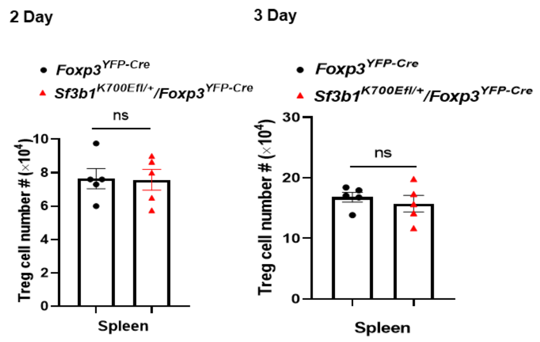

Fig. S1. A) RT-PCR analysis of WT and floxed-Sf3b1-K700E allele from *Sf3b1*$^{K700Efl/+}$/*Foxp3*$^{YFP-Cre}$ mice (left panel) or *Sf3b1*$^{K700Efl/+}$/*CD4*^{Cre} mice (right panel). **B**) Sequence analysis of Sf3b1-K700E (AAA-GAA) mutation using cDNA obtained from *Sf3b1*$^{K700Efl/+}$/*CD4*^{Cre} $CD4^+$ T cells. **C**) Thymic cellularity quantified by Cellometer (n=3-4 per genotype). **D**) Representative flow cytometric analysis of CD4 and CD8 on thymocytes from indicated mice. Right panel: summary of the percentage of $CD4^-CD8^-$ double negative (DN), $CD4^+CD8^+$ double positive (DP), CD4 single positive (SP) and CD8 single positive (SP) thymocytes shown on left (n⩾5 per genotype). **E)** Representative flow cytometric analysis (left panels) and percentage (right panel) of $Helios^+Foxp3^+$ $CD4^+$ cells and of $Nrp\text{-}1^+Foxp3^+$ $CD4^+$ cells of indicated mice (n⩾4 per genotype per group). **F)** Representative flow cytometric analysis (left panels) and percentage (right panel) of naive $Foxp3^-$ $CD4^+$ cells purified from spleens of indicated mice (n⩾4 per genotype per group). **G**) Representative flow cytometric analysis (left panels) and percentage (right panels) of live cells among indicated $CD4^+$ cells polarized for 48 hrs under T_{reg} conditions shown in Fig. 1D (n⩾4 per genotype per group). **H)** Representative flow cytometric analysis (left panels) and percentage (right panel) of naive $Foxp3^-CD4^+$ cells isolated from spleens of indicated mice (n = 4 per genotype). **I**) The number of indicated YFP^+ T_{regs} on day two or three after stimulation of 0.2 x 10^6 purified spleen T_{regs} from indicated mice in the presence of 20 ng/ml IL-2 (n ⩾ 4 per genotype). Boxed region: cell population of interest. Data are from three experiments (C, I ; D, E, F, G, H, right panels; presented as mean ± SEM) or are from one representative of three independent experiments (A, B; D, E, F, G, H, left panels). ns, not significant (two-tailed Students' t-test).

Fig. S2

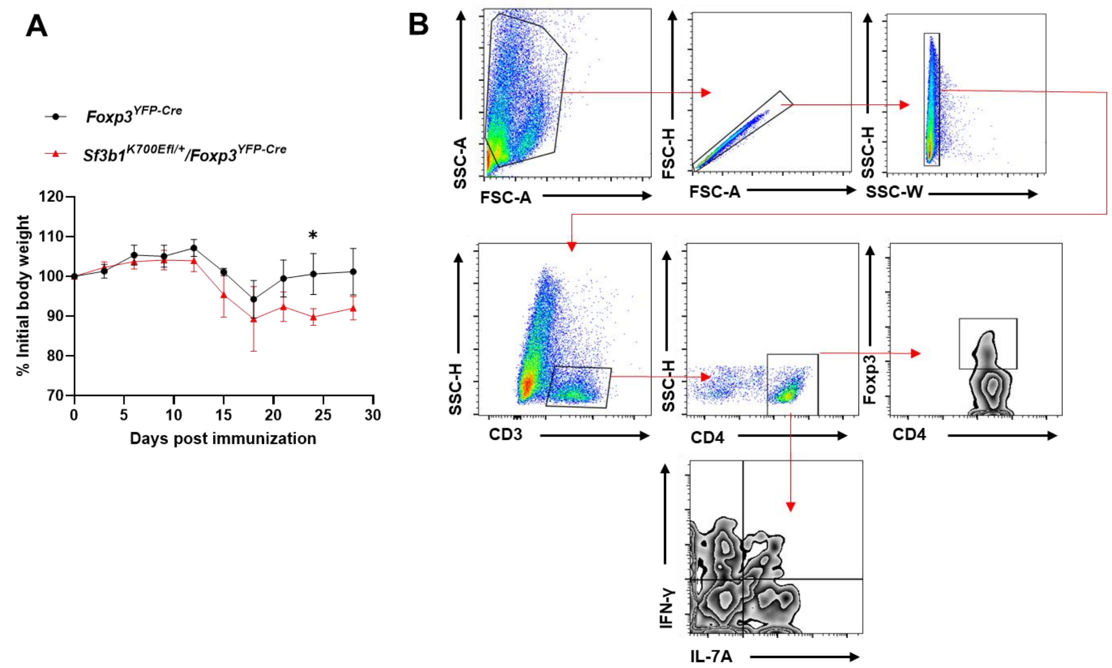

Fig. S2. A) Body weight of EAE-induced mice shown in Fig. 2C (n ⩾ 4 per genotype). **B**) Gating strategy for Fig. 2D and Fig. 2F. Boxed region: cell population of interest.

Fig. S3

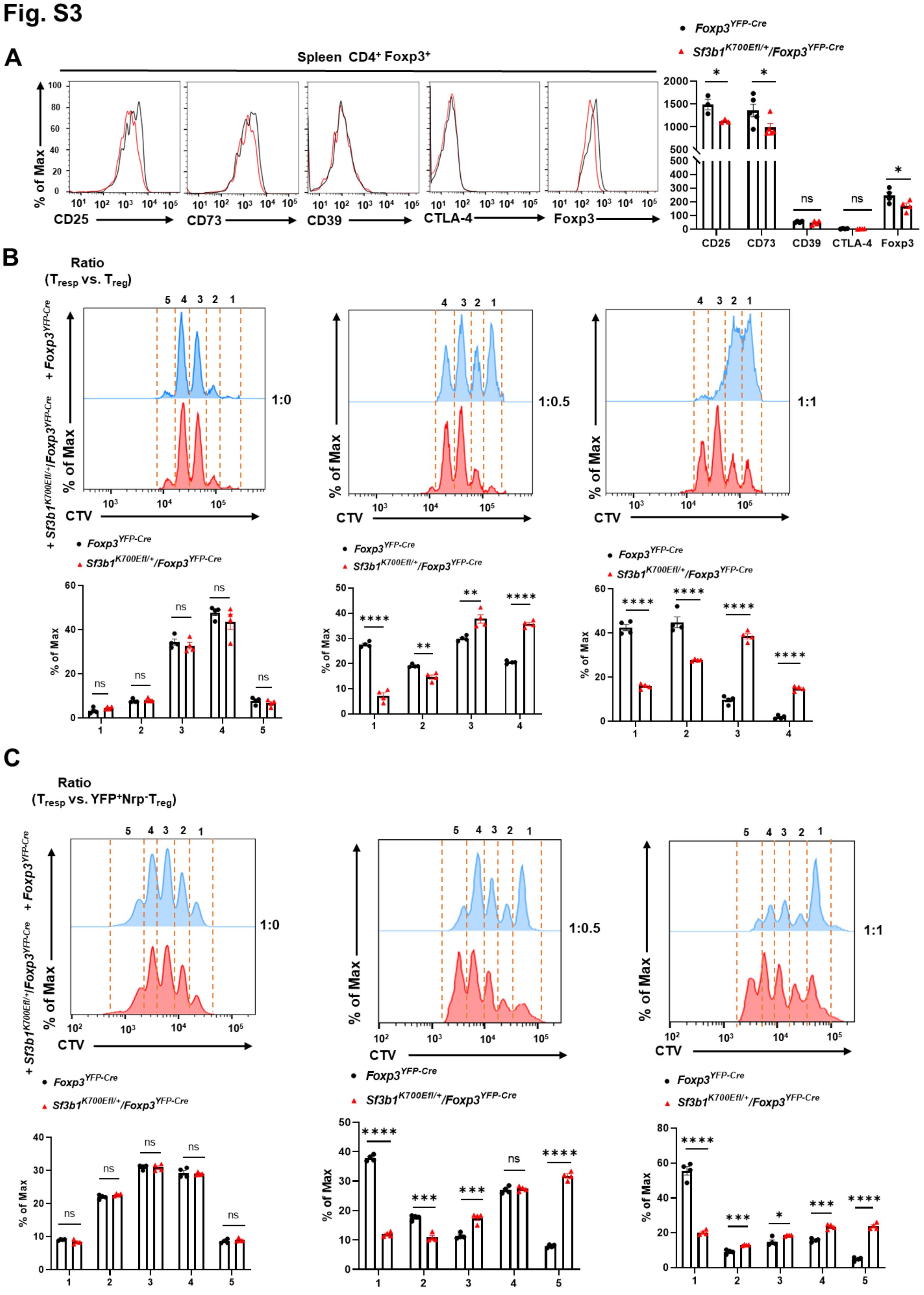
A
Spleen CD4+ Foxp3+
Foxp3YFP-Cre
Sf3b1K700Efl/+/Foxp3YFP-Cre
% of Max
CD25
CD73
CD39
CTLA-4
Foxp3
B
Ratio
(Tresp vs. Treg)
+ Foxp3YFP-Cre
+ Sf3b1K700Efl/+/Foxp3YFP-Cre
CTV
1:0
1:0.5
1:1
C
Ratio
(Tresp vs. YFP+Nrp-Treg)
ns
*
**
***
****

Fig. S3-continued

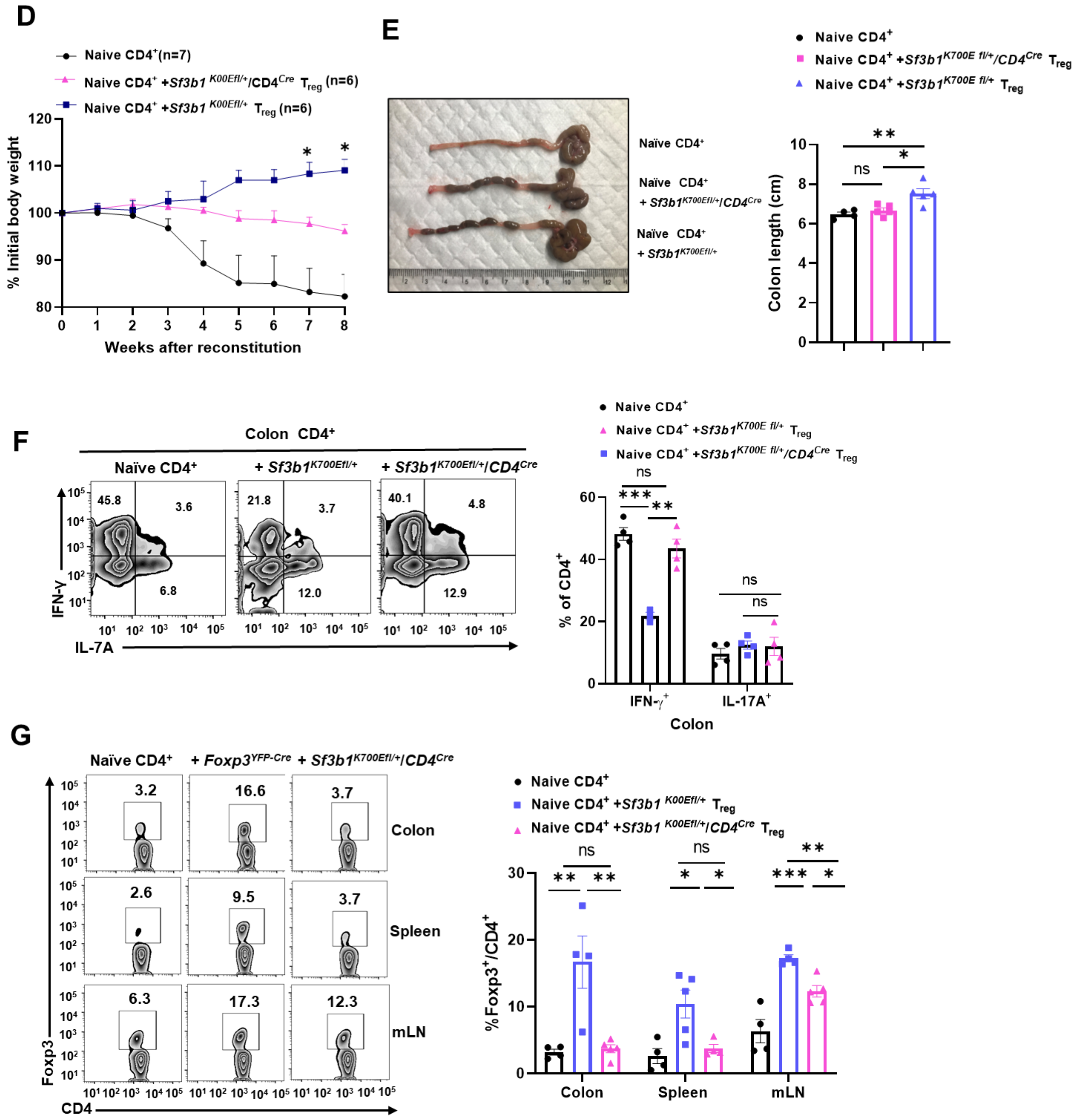

Fig. S3. A) Representative flow cytometric analysis (left panels) and the MFI (right panels) of T_{reg} surface markers (CD25, CD73, CD39, CD25 and CTLA-4) among $CD4^{+}Foxp3^{+}$ cells from the spleens of 8-10 weeks *Foxp3*$^{YFP\text{-}Cre}$ and *Sf3b1*$^{K700Efl/+/}$*Foxp3*$^{YFP\text{-}Cre}$ mice (n⩾4 per genotype). **B**) Representative flow cytometric analysis (top panels) and percentage (bottom panel) of proliferative dye-labelled responder $CD4^{+}$ T cells (T_{resp}), co-cultured with splenic $YFP^{+}CD4^{+}$ T_{regs} isolated from indicated 6- to 8-week-old mice as shown in Fig. 3A (n ⩾ 4 per genotype). **C**) Representative flow cytometric analysis (top panels) and percentage (bottom panel) of

proliferative dye-labelled responder $CD4^+$ T cells (T_{resp}), co-cultured with splenic YFP^+CD4^+ $Nrp\text{-}1^-$ T_{regs} isolated from indicated 6- to 8-week-old mice (n = 4 per genotype). **D**) Body weight of *Rag1*$^{-/-}$ recipients over time after adoptive transfer of naïve WT $CD45RB^{hi}CD25^-CD4^+$ T cells alone or in combination with splenic T_{regs} from 8- to 10-week-old *Sf3b1*$^{K700Efl/+}$ or *Sf3b1*$^{K700Efl/+/}$*CD4*^{Cre} mice. **E**) Image of colons (left panel) and colon length (right panel) from colitis-induced mice shown in D (n ⩾ 5 per genotype). **F**) Representative flow cytometric analysis (left panels) and percentage (right panel) of $CD4^+IL\text{-}17A^+$ and $CD4^+IFN\text{-}\gamma^+$ cells recovered from colons of colitis-induced recipients shown in D (n ⩾ 4 per group). **G**) Representative flow cytometric analysis (left panels) and percentage (right panel) of $CD4^+$ $Foxp3^+$ T_{regs} recovered from colon, spleen, and mLN of colitis-induced recipients shown in D (n ⩾ 4 per group). Boxed region: cell population of interest. Data are from three experiments (D ; B, C bottom panel; A, E, F and G, right panels; presented as mean ± SEM) or are from one representative of three independent experiments (B,C top panel; A, E, F and G, left panels). **P*<0.05; ***P*<0.01 ; ****P* < 0.001, and **** *P*<0.0005; ns, not significant (two-tailed Students' t-test).

Fig. S4

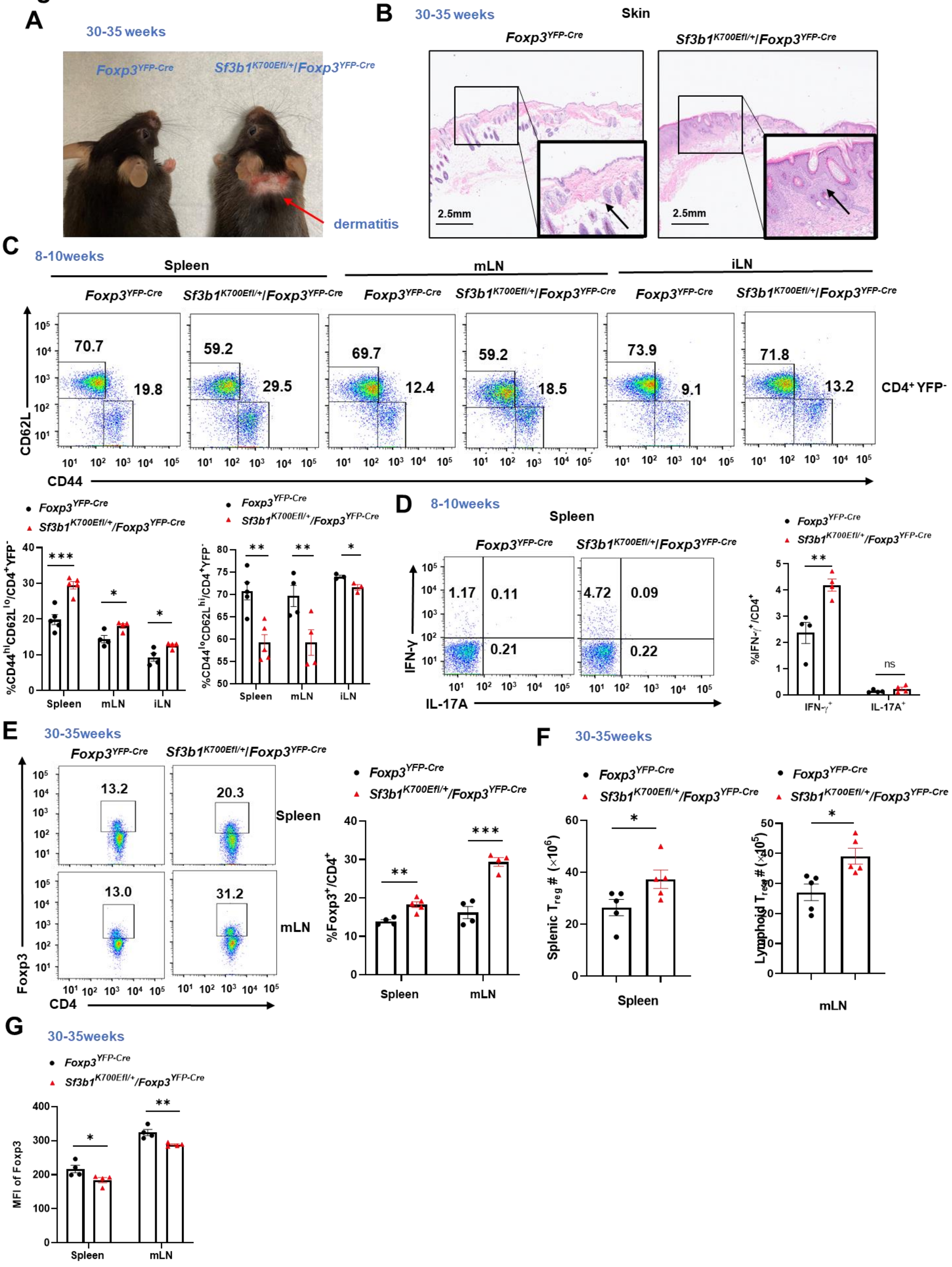
A
30-35 weeks
Foxp3YFP-Cre
Sf3b1K700Efl/+/Foxp3YFP-Cre
dermatitis
B
30-35 weeks
Skin
Foxp3YFP-Cre
Sf3b1K700Efl/+/Foxp3YFP-Cre
2.5mm
2.5mm
C
8-10weeks
Spleen
mLN
iLN
Foxp3YFP-Cre
Sf3b1K700Efl/+/Foxp3YFP-Cre
70.7
19.8
59.2
29.5
69.7
12.4
59.2
18.5
73.9
9.1
71.8
13.2
CD4+ YFP-
CD62L
CD44
%CD44hiCD62Llo/CD4+YFP-
%CD44loCD62Lhi/CD4+YFP-
Spleen
mLN
iLN
D
8-10weeks
Spleen
IFN-γ
IL-17A
1.17
0.11
0.21
4.72
0.09
0.22
%IFN-γ+/CD4+
IFN-γ+
IL-17A+
ns
E
30-35weeks
Spleen
mLN
13.2
20.3
13.0
31.2
Foxp3
CD4
%Foxp3+/CD4+
F
30-35weeks
Splenic Treg # (×10^6)
Lymphoid Treg # (×10^5)
G
30-35weeks
MFI of Foxp3

Fig. S4. A) Representative picture of aged *Foxp3*$^{YFP\text{-}Cre}$ and *Sf3b1*$^{K700Efl/+/}$*Foxp3*$^{YFP\text{-}Cre}$ mice to show hair loss. **B**) H&E-stained of skin section from aged *Foxp3*$^{YFP\text{-}Cre}$ and *Sf3b1*$^{K700Efl/+/}$*Foxp3*$^{YFP\text{-}Cre}$ mice. **C**) Representative flow cytometric analysis (top panels) and percentage (bottom panels) of $CD44^{hi}CD62^{lo}$ memory-like and $CD44^{lo}CD62^{hi}$ naive cells among splenic $CD4^+$ T cells from 8-10 weeks *Foxp3*$^{YFP\text{-}Cre}$ and *Sf3b1*$^{K700Efl/+/}$*Foxp3*$^{YFP\text{-}Cre}$ mice (n⩾4 per genotype). **D**) Representative flow cytometric analysis (left panels) and percentage (right panel) of $CD4^+$IL-$17A^+$ and $CD4^+$IFN-γ^+ cells recovered from spleen of 8-10 weeks old *Foxp3*$^{YFP\text{-}Cre}$ and *Sf3b1*$^{K700Efl/+/}$*Foxp3*$^{YFP\text{-}Cre}$ mice (n⩾4 per genotype per group). **E**) Representative flow cytometric analysis (left panel) and percentage of T_{regs} (right panel) among $CD4^+$ cells recovered from spleen and mesenteric LN (mLN) of indicated genotypes of aged mice (n⩾4 per genotype per group). **F**) Absolute number of T_{regs} among $CD4^+$ cells recovered from spleen and mLN of indicated genotypes of 30-35 weeks old mice (n⩾4 per genotype per group). **G**) MFI of Foxp3 from spleen and mLN of indicated genotypes of 30-35 weeks old mice (n⩾4 per genotype per group). Boxed region: cell population of interest. Data are from three experiments (B; C, bottom panels; D, right panels; presented as mean ± SEM), or are from one representative of three independent experiments (A, B; C, top panels; D, E, left panels). *P<0.05; **P<0.01, and ***P < 0.001; ns, not significant (two-tailed Students' t-test).

Fig. S5

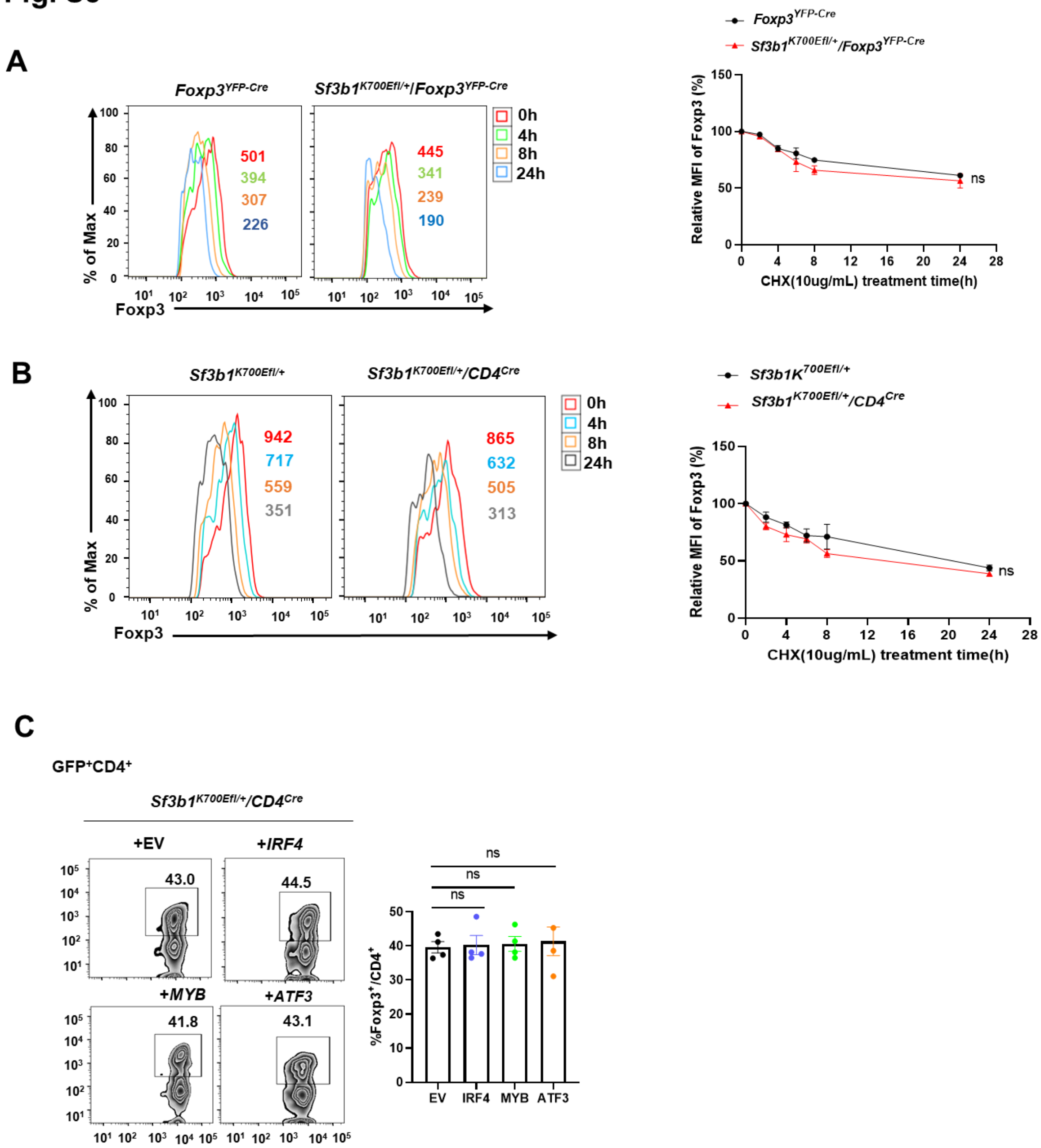

Fig. S5. A-B) Flow cytometric analysis of Foxp3 levels (left two panels) and relative degradation rate (right panel) in *in vitro* differentiated T_{regs} from *Foxp3*$^{YFP\text{-}Cre}$ and *Sf3b1*$^{K700Efl/+/}$*Foxp3*$^{YFP\text{-}Cre}$ $CD4^{+}$ T cells (A) or *Sf3b1*$^{K700Efl/+}$*/* and *Sf3b1*$^{K700Efl/+}$*/CD4*^{Cre} mice (B) and treated with protein synthesis inhibitor CHX for different times (n=4 per genotype). **C**) Representative flow cytometric analysis (left panel) and the percentage (right panel) of $Foxp3^{+}$ T_{regs} among *Sf3b1*$^{K700Efl/+}$*/CD4*^{Cre} $CD4^{+}$ cells transduced with virus expressing GFP alone (EV) or together with *Irf4* , *Myb* or *Atf3* and polarized under T_{reg} conditions for 48 hours (n⩾3 per treatment per group). Boxed region: cell population of interest. Data are from three experiments (A, B, C, right panels; presented as mean ± SEM) or are from one representative of three independent experiments (A, B, C, left panels). ns, not significant (two-tailed Students' t-test).

Fig. S6

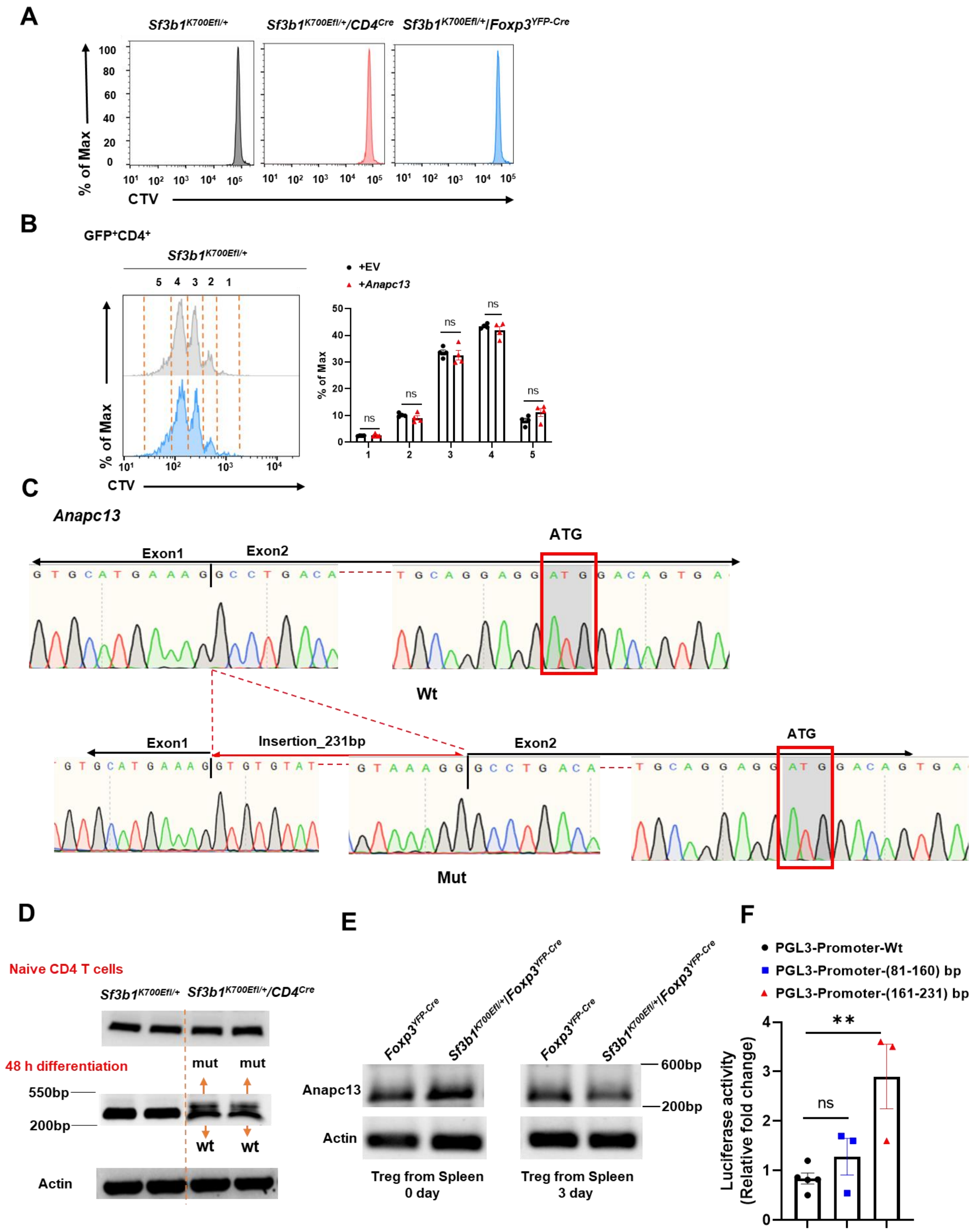
A
Sf3b1K700Efl/+
Sf3b1K700Efl/+/CD4Cre
Sf3b1K700Efl/+/Foxp3YFP-Cre
% of Max
CTV
B
GFP+CD4+
Sf3b1K700Efl/+
+EV
+Anapc13
% of Max
CTV
ns
C
Anapc13
Exon1
Exon2
ATG
Wt
Insertion_231bp
Mut
D
Naive CD4 T cells
Sf3b1K700Efl/+
Sf3b1K700Efl/+/CD4Cre
48 h differentiation
mut
wt
550bp
200bp
Actin
E
Foxp3YFP-Cre
Sf3b1K700Efl/+/Foxp3YFP-Cre
Anapc13
Actin
600bp
200bp
Treg from Spleen
0 day
Treg from Spleen
3 day
F
PGL3-Promoter-Wt
PGL3-Promoter-(81-160) bp
PGL3-Promoter-(161-231) bp
Luciferase activity
(Relative fold change)
ns
**

Fig. S6. A) Representative flow cytometric analysis of naïve $CD4^+$ cells from indicated mice after labelled with CTV prior to proliferation assay shown in Fig. 6B. **B**) Representative flow cytometric analysis (left panels) and the percentage (right panel) of indicated proliferative-dye-labelled GFP^+CD4^+ cells transduced with retrovirus expressing GFP alone (EV) or together with *Anapc13* and polarized for 48 hours under T_{reg} conditions (n $\geqslant$ 4 per genotype per group). **C**) Sequence of analysis of PCR amplified WT (wt) and alternatively spliced (mut) *Anapc13* transcript. **D**) RT-PCR analysis of WT (wt) and alternatively spliced (mut) *Anapc13* in indicated naive $CD4^+$ T cells (top panel) or differentiated T_{regs}. Actin (bottom panel) is a loading control. **E**) RT-PCR analysis of WT and alternatively spliced Anapc13 in indicated Tregs before and after stimulation of purified spleen Tregs for three days in the presence of IL-2 shown in supplementary Fig. 1I. **F**) Relative luciferase activity from a reporter described in Fig. 6G but with indicated portion of 231 bp insertion deleted. Data are from three experiments (B, right panels; F; presented as mean ± SEM) or are from one representative of three independent experiments (A,D,E; B, left panels). ***P*<0.01; ns, not significant (two-tailed Students' t-test).

Fig. S7

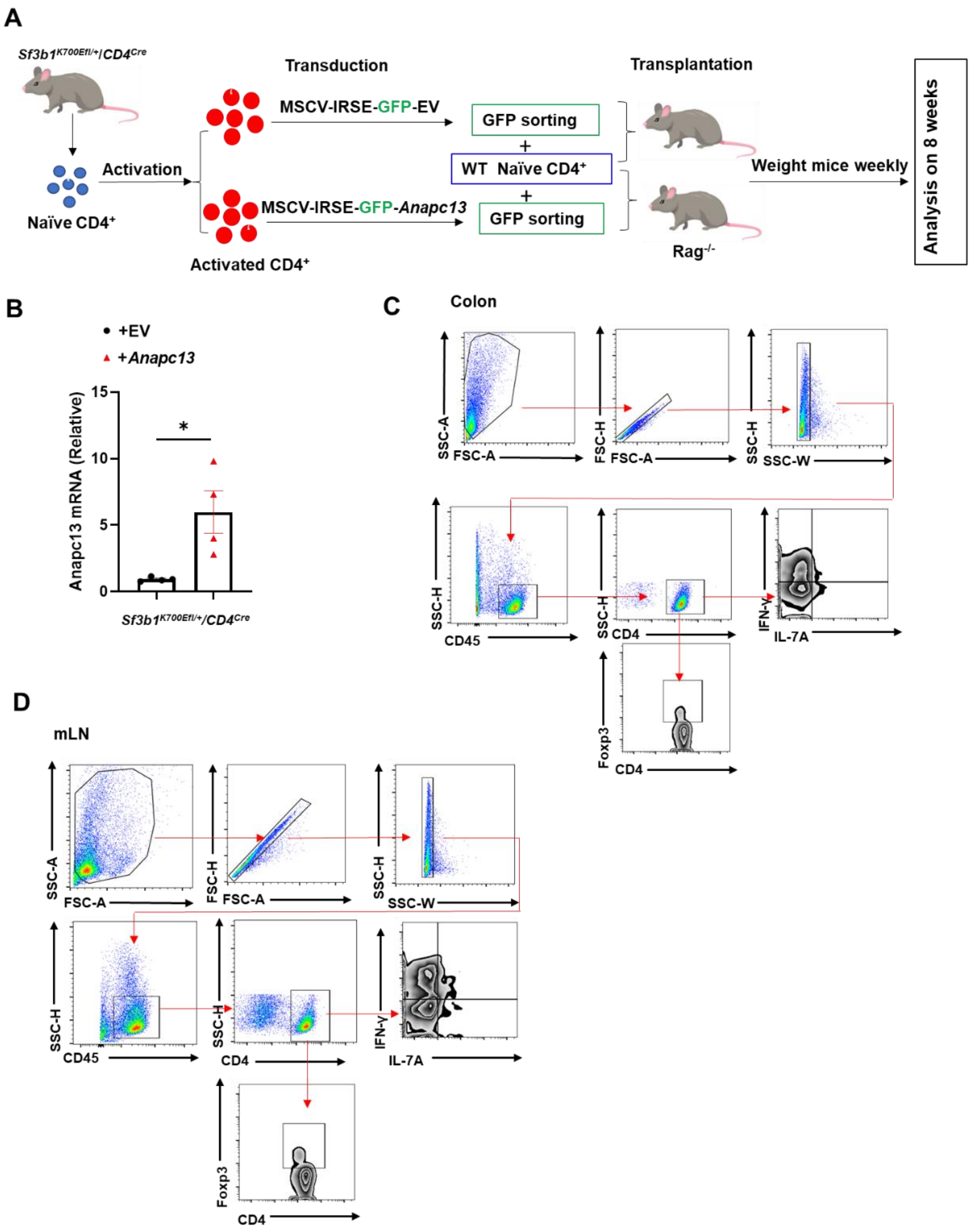

Fig. S7. A) Overview of the experimental procedure used for forced expression of *Anapc13* in *Sf3b1*$^{K700Efl/+}$/*CD4*^{Cre} T_{regs} to rescue colitis. **B**) qPCR analysis of *Anapc13* mRNA in *Sf3b1*$^{K700Efl/+}$/*CD4*^{Cre} T_{regs} retrovirally expressing GFP alone (EV) or with *Anapc13* (n ⩾ 4 per genotype per group). **C-D**) Gating strategy for Fig. 7D and E. *P<0.05. Boxed region: cell population of interest. Data are from three experiments (B; presented as mean ± SEM)

Fig.S8

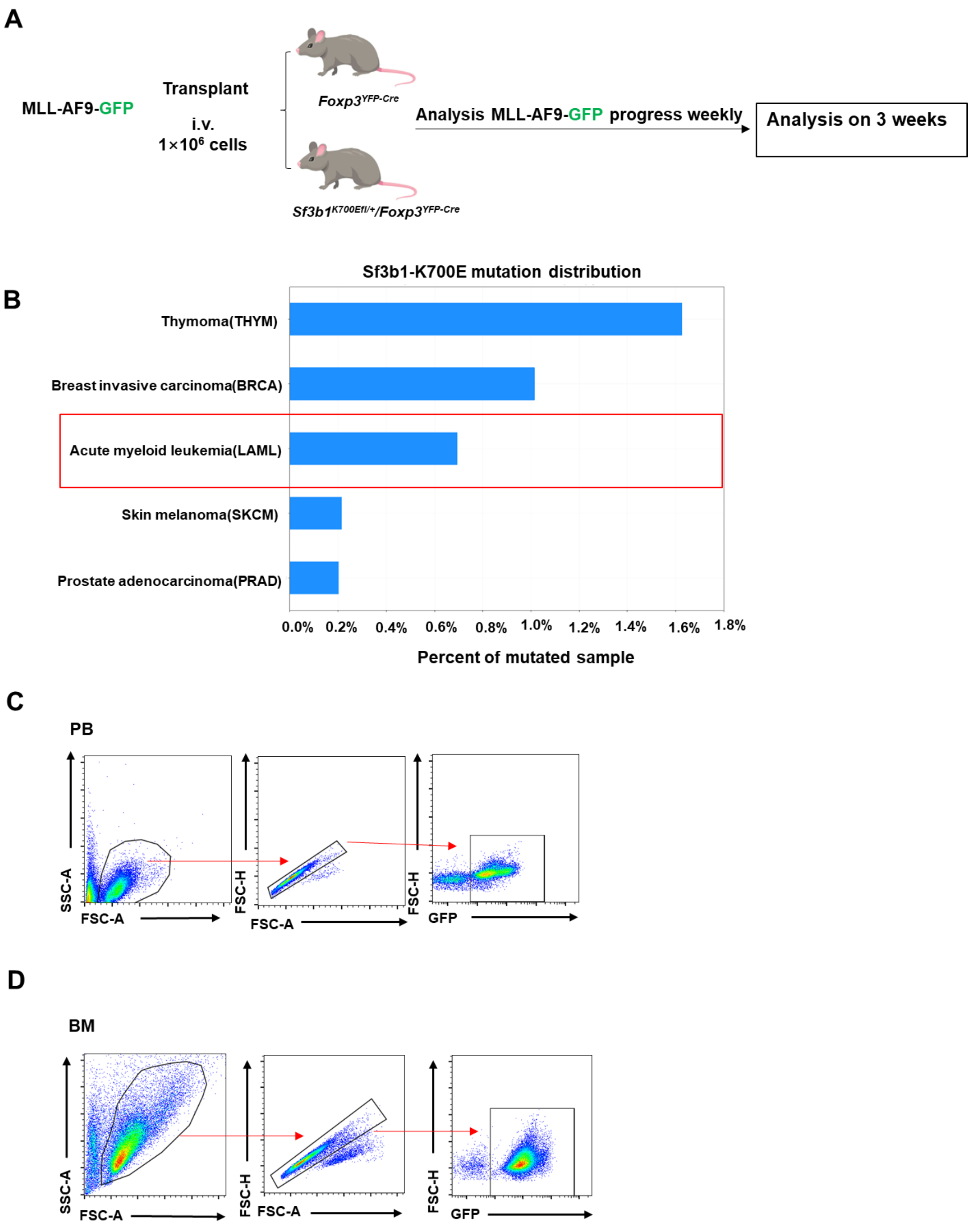

Fig. S8. A) Schematic of experimental design. GFP^{+} AML cells (1×10^{6}) were transplanted via tail vein i.v. injection into recipient mice to generate AML. The percentage of GFP^{+} cells in peripheral blood were monitored every week. **B**) SF3B1-K700E mutation distribution based on TCGA public cohort mining .**C-D**) Gating strategy for Fig. 8 D and E. Boxed region: cell population of interest.

Table S1. List of primers sequences used in this study.

qPCR	Gene	Forward	Reverse
	Foxp3	CCCATCCCCAGGAGTCTTG	ACCATGACTAGGGGCACTGTA
	Myb	AGACCCCGACACAGCATCTA	CAGCAGCCCATCGTAGTCAT
	Irf4	TCCGACAGTGGTTGATCGAC	CCTCACGATTGTAGTCCTGCTT
	Id1	GACATGAACGGCTGCTACT	AGGTCCCTGATGTAGTCGAT
	Atf3	GGAGTCAGTTACCGTCAACAA	CACTTGGCAGCAGCAATTT
	Anapc13	ATGGACAGTGAGGTACAGCG	TCAGTTTCCAGCGGGTGGGA
	Actin	GGGAAATCGTGCGTGACAT	GTCAGGCAGCTCGTAGCTCTT

RT-PCR	Gene	Forward	Reverse
	Anapc13	ATTTACAAAGCTGTGTGCA	TCAGTTTCCAGCGGGTGGGA
